# Short-Chain Fatty Acids at the Crossroads of Microbiota, Immunometabolism, and Inflammation

**DOI:** 10.3390/biomedicines14091967

**Published:** 2026-08-31

**Authors:** Łucja Rolek, Agata Sowa, Milena Czosnek, Ewelina Grywalska, Paulina Mertowska, Sebastian Mertowski

**Affiliations:** 1 Department of Experimental Immunology, Medical University of Lublin, 20-093 Lublin, Polandewelina.grywalska@umlub.edu.pl (E.G.); paulina.mertowska@umlub.edu.pl (P.M.); 2 Student Research Group of Experimental Immunology, Medical University of Lublin, 20-093 Lublin, Poland

**Keywords:** short-chain fatty acids, immunometabolism, histone deacetylase inhibitors, gut–immune axis, next-generation probiotics, microbiota-accessible carbohydrates, immune system

## Abstract

The rising incidence of chronic autoimmune and autoinflammatory diseases has been increasingly associated with environmental and lifestyle factors, including Western dietary patterns, intestinal dysbiosis, and reduced production of short-chain fatty acids (SCFAs). Reduced production of acetate, propionate, and butyrate has been associated with impaired epithelial barrier function, altered peripheral immune tolerance, and low-grade systemic inflammation. This article integrates and systematizes current knowledge in the field of immunometabolism, focusing on the role of the microbiota–metabolism–immunity axis. The molecular mechanisms by which these bacterial metabolites modulate immune function—both through the activation of specific surface receptors and direct epigenetic regulation—are analyzed in detail. SCFAs have been shown to actively reprogram the metabolic and transcriptional profiles of effector cells, stimulating anti-inflammatory macrophage polarization, suppressing cellular inflammatory cascades, and inducing the differentiation of protective regulatory T cells. To address the pharmacokinetic limitations of natural fatty acids, this study critically evaluates modern translational strategies. The clinical potential of synthetic receptor agonists, selective epigenetic modulators, and advanced next-generation bacterial consortia is analyzed. The presented data synthesis not only organizes the pathophysiological foundations but, above all, points to promising new directions for personalized non-pharmacological immunomodulation in the treatment of inflammatory diseases.

## 1. Introduction

The prevalence of autoimmune and autoinflammatory diseases has risen sharply in recent decades, representing a major challenge to public health, particularly in highly developed countries. Current estimates indicate that autoimmune diseases affect approximately 8–10% of the global population [1]. Despite their nomenclatural similarity, autoimmune and autoinflammatory diseases differ fundamentally in their etiology and pathogenesis. In autoimmune diseases, dysregulation of the adaptive immune response, driven primarily by T and B lymphocytes, plays a central role. A breakdown in self-tolerance mechanisms can result in autoreactive immune responses directed against host tissues [2]. Conversely, autoinflammatory diseases stem from impaired innate immunity, characterized by the uncontrolled activation of cells such as macrophages, neutrophils, and dendritic cells. This defect often results from genetic abnormalities in pattern-recognition receptors (PRRs) [3]. The rapid surge in immune-mediated disorders has prompted researchers to investigate their underlying environmental and lifestyle triggers. Accumulating evidence suggests that progressive urbanization and societal ‘westernization’ are associated with changes in immune function and with an increased risk of immune-mediated disorders [4]. Chief among these contemporary lifestyle modifications is a profound shift in dietary habits. Over the past few decades, traditional diets rich in fiber and plant-based foods have been largely supplanted by the Western diet, which is heavily based on simple sugars, ultra-processed foods, and a limited intake of fiber [5,6]. Nutritional patterns can influence macrophage activation, lymphocyte responses, and epithelial barrier integrity, in part through changes in the composition and functional activity of the gut microbiota [5]. Nearly 90% of this bacterial population consists of two phyla: Firmicutes (now also referred to as Bacillota) and Bacteroidetes (Bacteroidota) [7]. Minor phyla, including Actinobacteria, Proteobacteria, and Verrucomicrobiota constitute the remainder of the microbiome, the precise composition of which depends on age, medication history, and dietary habits [8]. In physiological conditions, the gut microbiota exists in a state of eubiosis, defined as a relatively stable and functionally balanced microbial ecosystem that supports host metabolic, epithelial, and immune homeostasis. In contrast, dysbiosis refers to a disruption of this microbial equilibrium, which may involve alterations in microbial diversity, the relative abundance of commensal and potentially pathogenic taxa, and, importantly, the functional output of the microbial community. Such disturbances may impair the production of beneficial microbial metabolites, including short-chain fatty acids, compromise epithelial barrier integrity, and promote inappropriate immune activation. Therefore, the transition from eubiosis to dysbiosis should be understood not only as a taxonomic shift in microbial composition, but also as a functional alteration of host–microbiota interactions that may contribute to the development or persistence of chronic inflammatory conditions [9,10,11,12,13]. One of the most critical functions of the microbiota is the anaerobic fermentation of fiber and non-starch polysaccharides, a process carried out primarily by commensal bacteria of the genera *Faecalibacterium*, *Roseburia*, *Eubacterium*, and *Bacteroides* [14]. The products of this process are short-chain fatty acids (SCFAs)—1-to-6 carbon saturated fatty acids that serve as key signaling mediators within the gut–immune axis [15]. Acetate (C2), propionate (C3), and butyrate (C4) are the most physiologically significant. They are found in the intestinal lumen in a ratio of approximately 60:20:20 [16]. Their functions and metabolic fates vary. For example, acetate enters the peripheral circulation as an energy substrate, propionate participates in hepatic gluconeogenesis; and butyrate serves as an energy source for colonocytes, while also exhibiting the strongest immunomodulatory effect [17,18]. SCFAs are increasingly recognized as important signaling metabolites capable of modulating inflammatory gene expression, intestinal barrier function, and leukocyte differentiation through receptor-dependent and receptor-independent mechanisms [19,20]. The anatomical and functional basis for this cellular communication is the gut-immune system axis. It is based on GALT (gut-associated lymphoid tissue), which constitutes the largest part of the human immune system [21]. Progressive dysbiosis, intestinal barrier dysfunction, and reduced SCFA availability may contribute to impaired immune tolerance and enhanced activation of effector immune cells [22]. A key element of this process is an imbalance between regulatory T cells (Tregs) and pro-inflammatory effector cell populations [23]. A reduced abundance of SCFA-producing commensal bacteria and the accompanying decrease in SCFA production have been associated with low-grade chronic inflammation [5]. In recent years, there has been growing interest in immunometabolism, defined as the interdependence between metabolic processes and the functioning of immune system cells [24]. It is now known that the metabolic changes occurring in immune cells are not merely a consequence of their activation, but actively participate in the regulation of the immune response. In response to various stimuli, these cells can undergo metabolic reprogramming, which determines their activation, differentiation, and functions [25]. For this reason, increasing attention is being paid to factors that can modulate the metabolic pathways of immune cells, as they may influence the course of inflammatory processes and the maintenance of immune homeostasis [26].

The aim of this review is to provide a comprehensive discussion of the role of immunometabolism in the regulation of the immune response, with particular emphasis on the importance of SCFAs as key mediators of communication between the microbiota, host cell metabolism, and immune system function. This review focuses on the mechanisms through which SCFAs influence the activation, differentiation, and effector and regulatory functions of immune cells, as well as their role in maintaining immune homeostasis, epithelial barrier integrity, and the control of chronic inflammation. Particular attention will be paid to how SCFAs, which are produced primarily through the bacterial fermentation of undigested carbohydrates and dietary fiber, interact with immune system cells via receptor-mediated and epigenetic mechanisms.

In this context, we will discuss the role of G protein-coupled receptors, such as GPR41, GPR43, and GPR109A, as well as the ability of selected SCFAs, particularly butyrate and propionate, to inhibit the activity of histone deacetylases. These mechanisms may lead to changes in the expression of genes associated with the inflammatory response, immune tolerance, the differentiation of regulatory T cells, cytokine production, and the function of antigen-presenting cells. Another key objective of this review is to present SCFAs as metabolites with immunomodulatory effects whose influence is not limited to the local intestinal environment. SCFAs can interact with epithelial cells, macrophages, dendritic cells, neutrophils, T and B lymphocytes, as well as with distant organs and tissues via the systemic circulation.

Beyond their local effects within the gastrointestinal tract, these metabolites may also participate in interorgan communication, including signaling along the microbiota–gut–brain axis, thereby extending their potential biological relevance to systemic and neuroimmune processes [27]. Accordingly, this review aims to present SCFAs as components of the microbiota–metabolism–immunity axis that may play a role in regulating inflammatory and autoimmune responses, as well as in maintaining tolerance to environmental and commensal antigens.

## 2. Materials and Methods

### Literature Search and Selection Strategy

This narrative review was based on a structured literature search conducted in PubMed/MEDLINE and Web of Science. The search focused on studies addressing short-chain fatty acids in the context of gut microbiota, immune regulation, immunometabolism, epigenetic mechanisms, and autoimmune and autoinflammatory diseases. Search terms included combinations of “short-chain fatty acids”, “SCFA”, “acetate”, “propionate”, “butyrate”, “gut microbiota”, “immunometabolism”, “immune regulation”, “histone deacetylase”, “HDAC”, “FFAR2”, “GPR43”, “FFAR3”, “GPR41”, “GPR109A”, “T cells”, “B cells”, “macrophages”, “dendritic cells”, “neutrophils”, “natural killer cells”, “autoimmune diseases”, and “autoinflammatory diseases”.

Peer-reviewed original research articles, including in vitro studies, animal experiments, ex vivo studies, observational human studies, and clinical intervention studies, were considered together with systematic reviews, meta-analyses, and selected narrative reviews providing relevant mechanistic or clinical context. Priority was given to original studies when specific molecular, cellular, metabolic, or clinical effects were discussed, whereas review articles were used primarily to provide broader conceptual context and to identify additional relevant primary studies.

Articles were selected on the basis of their relevance to the microbiota–SCFA–immune axis, mechanistic contribution, methodological quality, and relevance to the clinical scope of the review. Particular attention was given to studies distinguishing the biological effects of acetate, propionate, and butyrate and to evidence describing concentration-, cell type-, tissue-, and disease-dependent effects of SCFAs. Because this article was designed as a narrative rather than a systematic review, no formal meta-analysis or quantitative evidence synthesis was performed.

## 3. The Effect of SCFAs on Immune Cells

### 3.1. Characteristics of SCFAs

**Figure 1:**
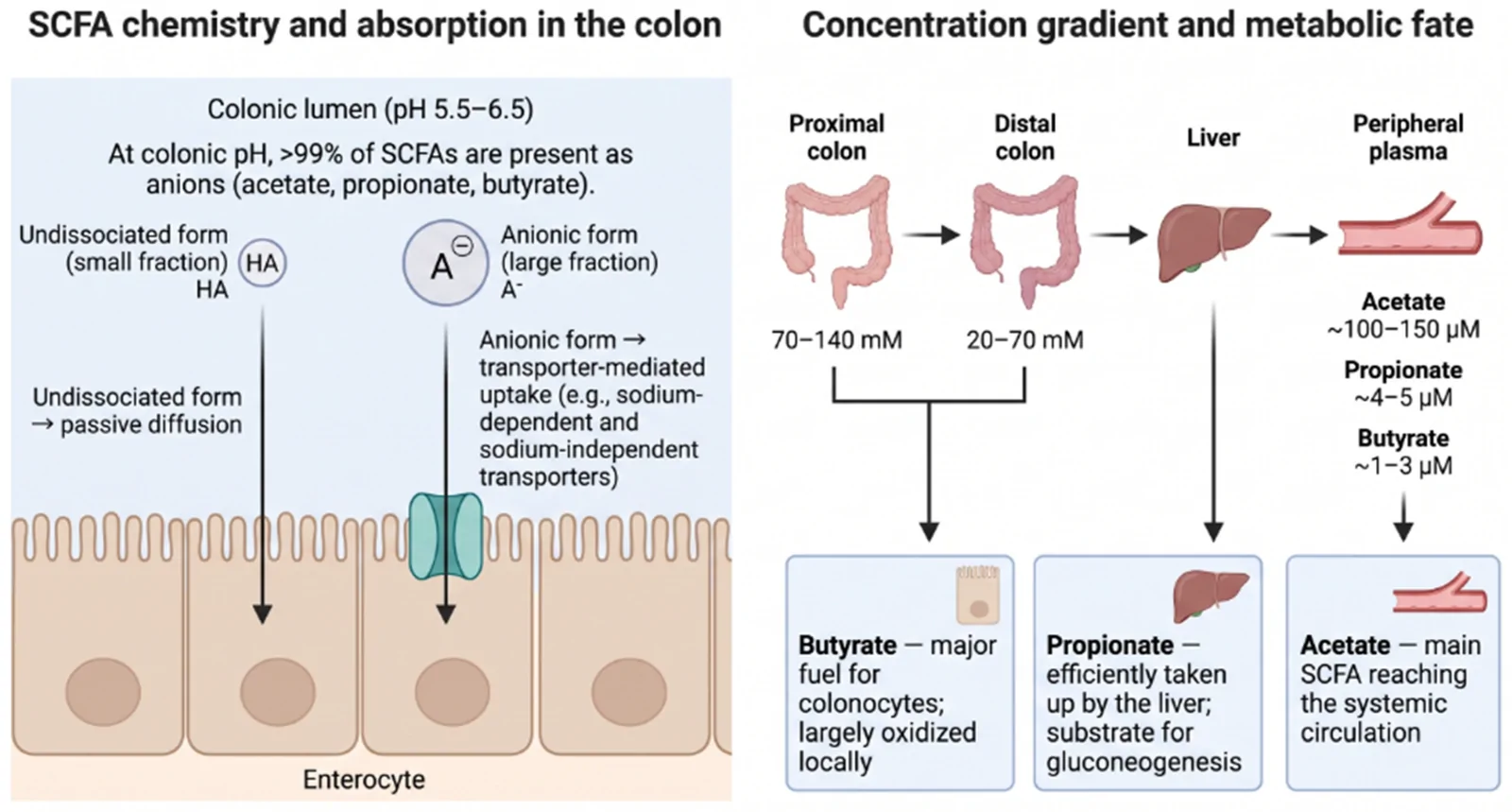
Colonic absorption, concentration gradient, and metabolic fate of the major SCFAs [14,15,16,17,18,28,29,30,31,32,33]. SCFAs are predominantly present as anions in the colonic lumen and are absorbed through passive diffusion and transporter-mediated mechanisms. Butyrate is largely utilized by colonocytes, propionate is preferentially taken up by the liver, whereas acetate represents the major SCFA reaching the peripheral circulation. Created in BioRender. Mertowski, S. (2026) https://BioRender.com/drcpafg (accessed on 10 July 2026).

SCFAs are a subgroup of saturated monocarboxylic acids characterized by the presence of an aliphatic carbon chain containing one to six carbon atoms [28]. This group includes formic acid (C1), acetic acid (C2), propionic acid (C3), butyric acid (C4), valeric acid (C5), and caproic acid (C6), as well as their branched-chain fatty acid (BCFA) counterparts, such as isobutyric acid and isovaleric acid [29]. Physicochemically, these molecules exhibit a low molecular weight and high aqueous solubility, which distinguishes them from long-chain fatty acids (LCFAs) and directly determines their unique pharmacokinetic properties in the human body [14]. Under physiological conditions within the large intestinal lumen (pH 5.5–6.5), SCFAs exist almost exclusively (>99%) as anions, such as acetate, propionate, and butyrate [30]. The degree of dissociation of these acids is closely related to their dissociation constant (pKa), which is approximately 4.8 for most SCFAs. Their ionization state is crucial for their bioavailability and how they penetrate biological barriers. Undissociated, or lipophilic, forms can cross the cell membranes of enterocytes by simple passive diffusion, whereas anionic forms require the involvement of specialized transport systems, including both sodium-gradient-dependent and sodium-gradient-independent systems [31]. An important aspect of SCFA biology is their distinct concentration gradient across different anatomical regions of the body. Depending on the diet, total SCFA concentrations can range from 70 to 140 mM in the proximal (ascending) colon and from 20 to 70 mM in the distal (descending) colon [16]. In the large intestine, they serve not only as a key source of energy but also as a direct modulator of the intestinal microenvironment. Most of the butyrate produced is immediately oxidized in colonocytes, while propionate is efficiently taken up by hepatocytes as a substrate for gluconeogenesis [32]. As a result, acetate is the main compound entering the peripheral circulation, and SCFA concentrations in peripheral blood plasma drop dramatically, reaching micromolar levels (approximately 100–150 μM for acetate, 4–5 μM for propionate, and 1–3 μM for butyrate) [33]. Despite such low peripheral concentrations, these molecules play an important role in metabolic modulation and the regulation of the immune response. Although acetate, propionate, and butyrate are commonly grouped as short-chain fatty acids, they are not biologically interchangeable. They differ substantially in their intestinal abundance, metabolic fate, systemic availability, receptor engagement, and epigenetic potency, which contributes to distinct and context-dependent effects on host metabolism and immune regulation [16,32,34,35,36,37]. The principal differences among the three major SCFAs are summarized in Table 1 and Figure 1.

### 3.2. The Molecular and Dual-Pathway Mechanism of Action of SCFAs as HDAC Inhibitors

Epigenetic modifications are heritable changes in gene expression and chromatin transcriptional activity. They occur without altering the nucleotide sequence of DNA itself. The basic mechanisms of this type include, among others, histone modification, which directly influences the structure of the cell nucleus, morphogenesis, and the course of physiological processes in cells [40]. The process of histone acetylation is reversible and involves two groups of enzymes with opposing functions: histone acetyltransferases (HATs), which stimulate transcription by loosening the chromatin structure, and histone deacetylases (HDACs). HDACs play a role in regulating gene transcription by catalyzing the removal of acetyl groups from lysine residues on core histones, as well as from non-histone proteins (such as p53 or NF-κB). This action allows them to control many biological processes [41]. The removal of acetyl groups leads to the condensation of the DNA structure and, consequently, to the silencing of gene expression [34]. Dysregulation of this epigenetic balance can induce aberrant gene expression in immune cells, potentially triggering autoimmune pathogenesis [40]. Contemporary research is increasingly focused on identifying environmental factors capable of modulating the host’s immune and metabolic responses. Particular importance is attributed to SCFAs, which are produced mainly through the bacterial fermentation of undigested dietary components, especially fiber. SCFAs are currently recognized as important mediators of the microbiota–metabolism–immunity axis, as they link the activity of the gut microbiota to the regulation of epithelial cell function, immune cell function, and inflammatory processes [35,36]. Their immunomodulatory effects are multifaceted and involve both rapid receptor-mediated mechanisms and slower epigenetic mechanisms associated, among other things, with the inhibition of histone deacetylase activity [35]. One of the main mechanisms of action of SCFAs is the activation of surface G protein-coupled receptors, such as FFAR2/GPR43, FFAR3/GPR41, and HCAR2/GPR109A. These receptors are present, among other places, on intestinal epithelial cells and immune system cells, where, upon binding to ligands, they trigger cascading intracellular signaling pathways that lead to the regulation of cAMP levels, the mobilization of calcium ions, the activation of MAPK/ERK pathways, and the modulation of cytokine and chemokine production [35,36]. FFAR2 and FFAR3 are classic SCFA receptors, whereas HCAR2, although it exhibits high affinity for niacin and D-β-hydroxybutyrate, also plays an important role in the anti-inflammatory effects of butyrate [36]. All three receptors belong to the GPCR family, displaying a characteristic seven-transmembrane domain architecture. Beyond their surface expression, their biological significance is dictated by distinct ligand profiles, tissue distribution, and specific immunoregulatory roles. The characteristics of the most important SCFA receptors are presented in Table 2.

The biological effects of SCFAs depend on the identity and concentration of the metabolite, tissue microenvironment, immune-cell type and activation state, receptor expression, host metabolic status, and disease context [16,37,38,39,45]. These determinants and their potential influence on SCFA-mediated immune regulation are summarized in Table 3.

**Figure 2:**
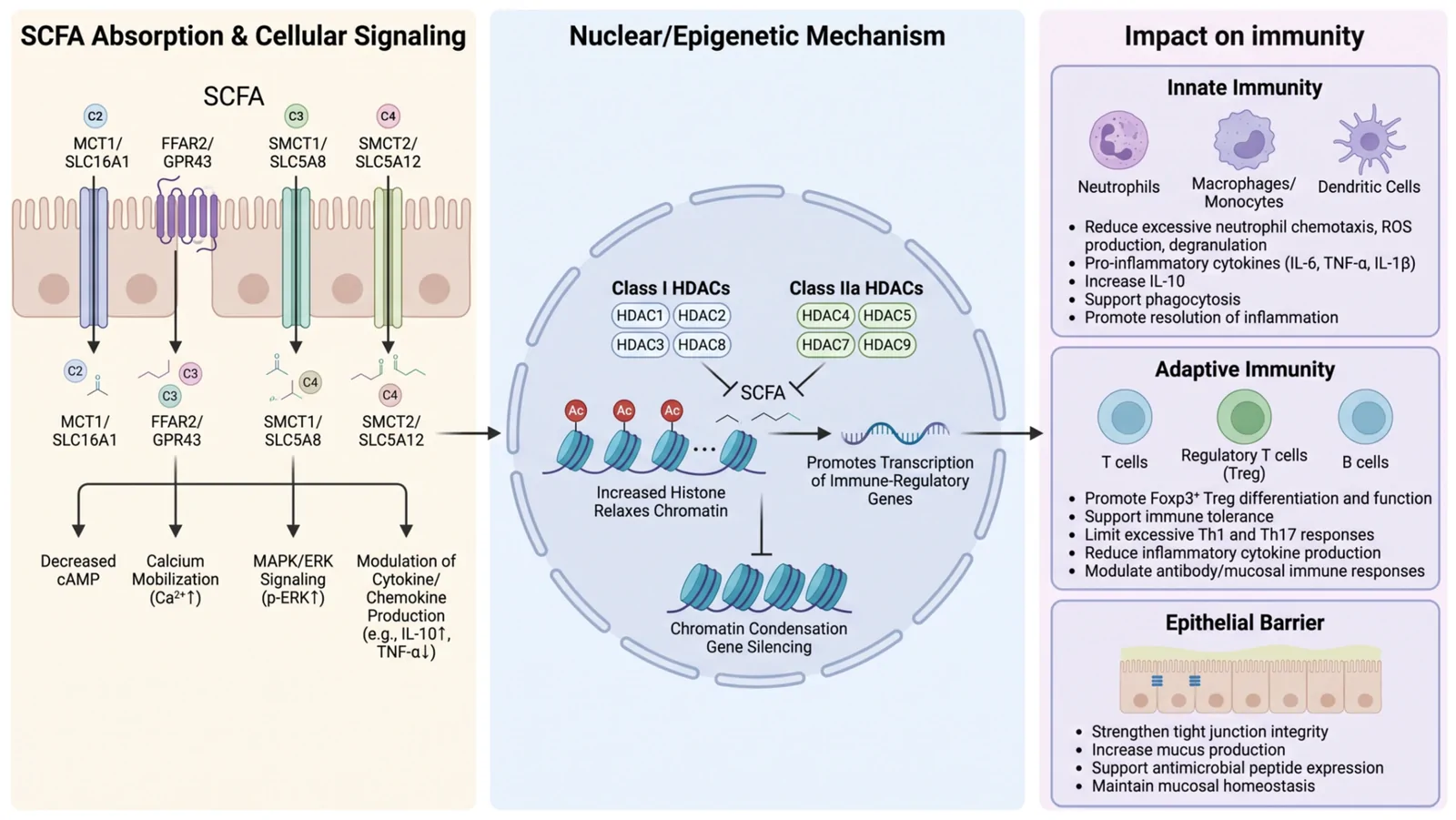
Major cellular mechanisms of SCFA signaling [34,35,36,40,41]. SCFAs influence epithelial and immune cells through receptor-dependent pathways and HDAC inhibition, thereby modulating immune responses and epithelial homeostasis in a context-dependent manner. ↑ indicates an increase; ↓ indicates a decrease. Created in BioRender. Mertowski, S. (2026) https://BioRender.com/drcpafg (accessed on 10 July 2026).

The expression of SCFA receptors varies by cell type and tissue, which determines the different scope of their biological effects. FFAR2/GPR43 plays a particularly important role in cells of the innate immune system, especially neutrophils, monocytes, and macrophages, whereas FFAR3/GPR41 links SCFA signaling to metabolic, neuroendocrine, and intestinal responses. HCAR2/GPR109A, on the other hand, functions as an anti-inflammatory receptor that is important for the effects of butyrate, niacin, and D-β-hydroxybutyrate, particularly in the context of neutrophil regulation, the epithelial barrier, and lipid metabolism [35]. Together, these receptors enable the translation of signals from the microbiota and diet into the regulation of immune homeostasis, the mucosal response, and chronic inflammation. On the other hand, SCFAs also exhibit direct intracellular activity that is independent of surface receptors. Among their receptor-independent mechanisms, butyrate and propionate can modulate histone deacetylase activity, with the best-characterized effects involving Class I HDACs, while additional interactions with Class IIa HDAC-dependent transcriptional programs have also been proposed [35,36]. This modulation can increase histone acetylation, alter chromatin accessibility, and influence the expression of genes involved in inflammation, immune tolerance, and mucosal homeostasis. In the context of immunometabolism, these effects provide an important mechanistic link between microbiota-derived metabolites and the transcriptional programming of host immune cells. Selected HDACs relevant to SCFA-mediated immune regulation are summarized in Table 4 and Figure 2.

Most SCFAs in the gastrointestinal tract are present as ions, which are absorbed in the cecum and colon in three ways: via monocarboxylate transporter 1 (MCT1/SLC16A1), via sodium-coupled monocarboxylate transporter 1 (SMCT1/SLC5A8), and by passive diffusion. SCFAs can also be transported into cells via the low-affinity transporter SMCT2 (SLC5A12), which mediates the absorption of molecules derived from the diet [43]. In terms of the dose–response relationship, butyrate is more effective than acetate and propionate at reducing the levels of pro-inflammatory mediators in various immune cell populations, which is a direct result of its highest affinity for the zinc-dependent active site of deacetylases [34]. However, its high biochemical efficacy does not imply nonspecific action across the entire genome—quite the contrary. The effect of butyrate is characterized by high selectivity. It has been shown that intracellular HDAC inhibition induced by butyrate modifies the expression of only 2% of mammalian genes. The promoters of these genes contain specific regulatory sequences, and butyrate itself acts by blocking the deacetylases HDAC1 and HDAC2 recruited by the transcription factors Sp1 and Sp3 [54].

### 3.3. Modulation of Innate Immune Cells and Antigen-Presenting Cells

**Figure 3:**
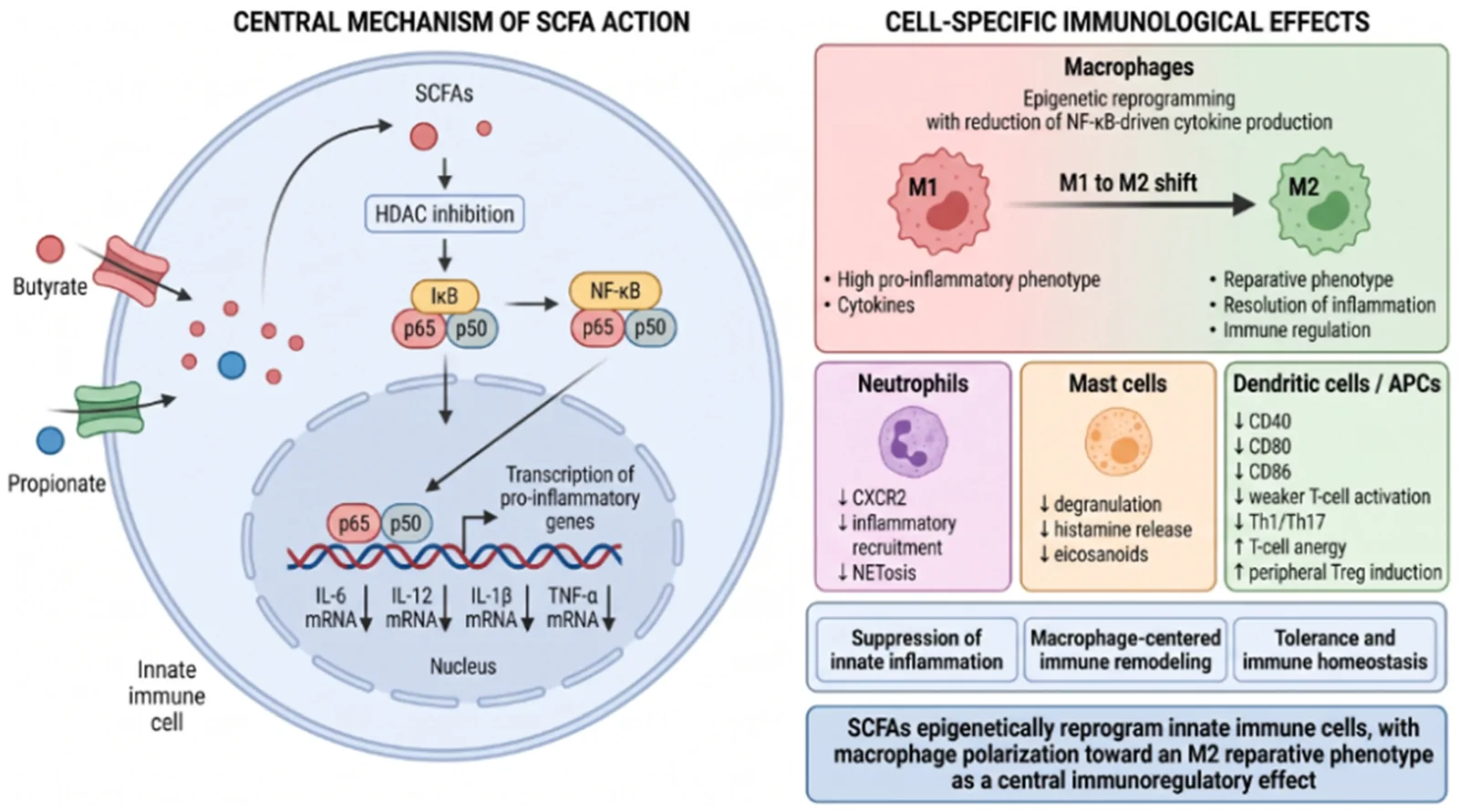
Experimental effects of SCFAs on innate immune-cell signaling [38,55,56,57,58,59]. SCFAs, particularly butyrate and propionate, can modulate HDAC- and NF-κB-associated pathways and influence macrophage, neutrophil, mast-cell, and dendritic-cell functions under selected experimental conditions. ↑ indicates an increase; ↓ indicates a decrease. Created in BioRender. Mertowski, S. (2026) https://BioRender.com/drcpafg (accessed on 10 July 2026).

At the level of the innate immune response, SCFAs—particularly butyrate and propionate—act as key regulators of the phenotypic plasticity of macrophages and dendritic cells (DCs). Experimental studies indicate that SCFA-mediated HDAC inhibition can modulate inflammatory transcriptional programs in innate immune cells; however, the magnitude and direction of these effects depend on the cell type and experimental setting [55,56,57]. This mechanism is based on the epigenetic modulation of the NF-κB (nuclear factor kappa-light-chain-enhancer of activated B cells) transcriptional axis. Evidence from experimental colitis and in vitro co-culture models indicates that butyrate can attenuate NF-κB-dependent inflammatory signaling and reduce the expression of pro-inflammatory mediators, including IL-6, IL-1β, and TNF-α [46,56,57] (Figure 3). Experimental evidence suggests that SCFAs may modulate neutrophil recruitment and NET formation and may also influence mast-cell activation and degranulation; however, these effects remain strongly dependent on the experimental context and SCFA concentration [58,59]. Furthermore, the epigenetic reprogramming of antigen-presenting cells (APCs) under the influence of SCFAs redefines their ability to initiate an adaptive immune response. Inhibition of histone deacetylases in maturing dendritic cells induces a state of resistance to activating signals (endotoxin tolerance), resulting in a drastic reduction in the expression of costimulatory molecules such as CD40, CD80, and CD86 on their surface. This impairs the immune synapse formed with naive T cells. As a result, SCFAs promote the formation of so-called tolerogenic dendritic cells (tDCs), which, instead of inducing the proliferation of Th1/Th17 effector lymphocytes, favor the anergy of autoreactive lymphocytes or their conversion into peripheral regulatory lymphocytes [38].

### 3.4. Epigenetic Reprogramming of the Adaptive Immune Response (T Cells and NK Cells)

Changes in the cytokine profile of antigen-presenting cells directly affect the cellular immune response. In experimental studies, butyrate and propionate were shown to suppress antigen-specific CD8+ T-cell activation, whereas acetate did not exert a comparable effect. Mechanistically, this effect was attributed primarily to reduced IL-12 production by antigen-presenting cells rather than to a direct suppressive action of these metabolites on CD8+ T cell [38]. However, the situation changes when SCFAs interact directly with intracellular HDAC enzymes within the lymphocytes themselves. As Park et al. indicate in their studies, SCFAs promote the differentiation of T lymphocytes into both effector and regulatory cells, stimulating an immune response or immune tolerance, respectively, depending on the current tissue conditions [45]. This context-dependent mechanism operates at the molecular level; SCFA-induced HDAC inhibition drives a global increase in histone acetylation across genes encoding pivotal transcription factors. This promotes the concurrent expression of the regulatory marker Foxp3 and effector lineage drivers, thereby adapting the final lymphocyte phenotype to signals from the surrounding inflammatory environment [45]. In CD4+ lymphocytes, SCFA-induced HDAC inhibition leads to the silencing of transcriptional programs responsible for the development of pro-inflammatory cell lineages, thereby suppressing the expression of the Ifng gene (for IFN-γ) in Th1 cells and the IL17 gene in pathogenic Th17 cells [60]. In vitro studies have shown that butyrate can enhance Foxp3 expression while attenuating RORγt-associated inflammatory transcriptional programs during T-cell differentiation. These findings provide mechanistic support for further investigation of SCFA-mediated modulation of the Treg/Th17 balance, although their clinical relevance remains to be established [60]. Conversely, when it directly affects CD8+, intracellular HDAC inhibition by butyrate induces epigenetic changes that optimize cellular metabolism and promote the formation of long-lived memory T cells with enhanced effector potential. Experimental studies have shown that SCFA-mediated metabolic and epigenetic reprogramming can enhance selected CD8+ T-cell effector functions, including granzyme B production, and may improve antitumor activity under specific experimental conditions [61]. Butyrate and pentanoate have also been reported to modulate CD8+ T-cell metabolism and differentiation toward phenotypes associated with enhanced persistence and effector potential in experimental models [61]. A similar mechanism of epigenetic regulation is observed in NK cells. In this cell population, SCFAs directly influence the expression of activating receptors and the production of interferon-gamma by modulating histone acetylation levels, which in turn modulates their cytotoxic properties in an inflammatory microenvironment [34].

### 3.5. Epigenetic and Metabolic Reprogramming of B Cells and the Humoral Immune Response

In addition to their previously described effect on the cellular immune response, SCFAs also play a key role in modulating humoral immune mechanisms. This is because the processes of differentiation, maturation, and effector function of B lymphocytes are subject to strict epigenetic and metabolic regulation controlled by metabolites of the gut microbiome [62]. At the transcriptional level, it has been shown that butyrate regulates the expression of many key genes essential for B-cell development and functional plasticity, with Aicda, Xbp1, and Prdm1 playing pivotal roles [53]. Experimental evidence indicates that SCFAs can influence B-cell differentiation and antibody responses, including enhanced IgA production and modulation of total and antigen-specific IgE responses [62]. Experimental studies have also implicated HDAC inhibition and p38 MAPK signaling in SCFA-mediated promotion of IL-10-producing regulatory B-cell phenotypes [63].

## 4. Immunometabolism: The Balance Between Glycolysis and Fatty Acid Oxidation

### 4.1. General Principles of Immunometabolism in Immune Cells

In recent years, increasing attention has been paid to the phenomenon of immunometabolism, defined as the interaction between metabolic pathways and the effector functions of immune system cells [64]. Emerging evidence indicates that cellular metabolism extends beyond simple bioenergetics, acting as a pivotal regulator of immune cell activation, differentiation, and survival of immune cells [65]. Distinct leukocyte lineages possess unique metabolic signatures that directly dictate their functional fates. Activated effector cells, such as Th1 and Th17 lymphocytes and cytotoxic NK cells, as well as pro-inflammatory M1 macrophages and mature dendritic cells, exhibit increased energy requirements and preferentially utilize glycolysis, even under conditions of normal oxygen availability [66,67]. This phenomenon, known as the Warburg effect, enables the rapid production of adenosine triphosphate (ATP) and the precursors necessary for the synthesis of proteins, lipids, and nucleotides that support the inflammatory response [68]. In contrast, a different metabolic profile is observed in regulatory and memory cells. Treg cells, memory T cells, anti-inflammatory M2 macrophages, and tolerogenic dendritic cells prefer oxidative phosphorylation (OXPHOS) and fatty acid oxidation (FAO). This allows them to function effectively in the microenvironment of chronic inflammation, which is characterized by a deficiency of oxygen and nutrients [69].

### 4.2. Molecular Mechanisms of SCFA-Mediated Modulation of Immunometabolism

**Figure 4:**
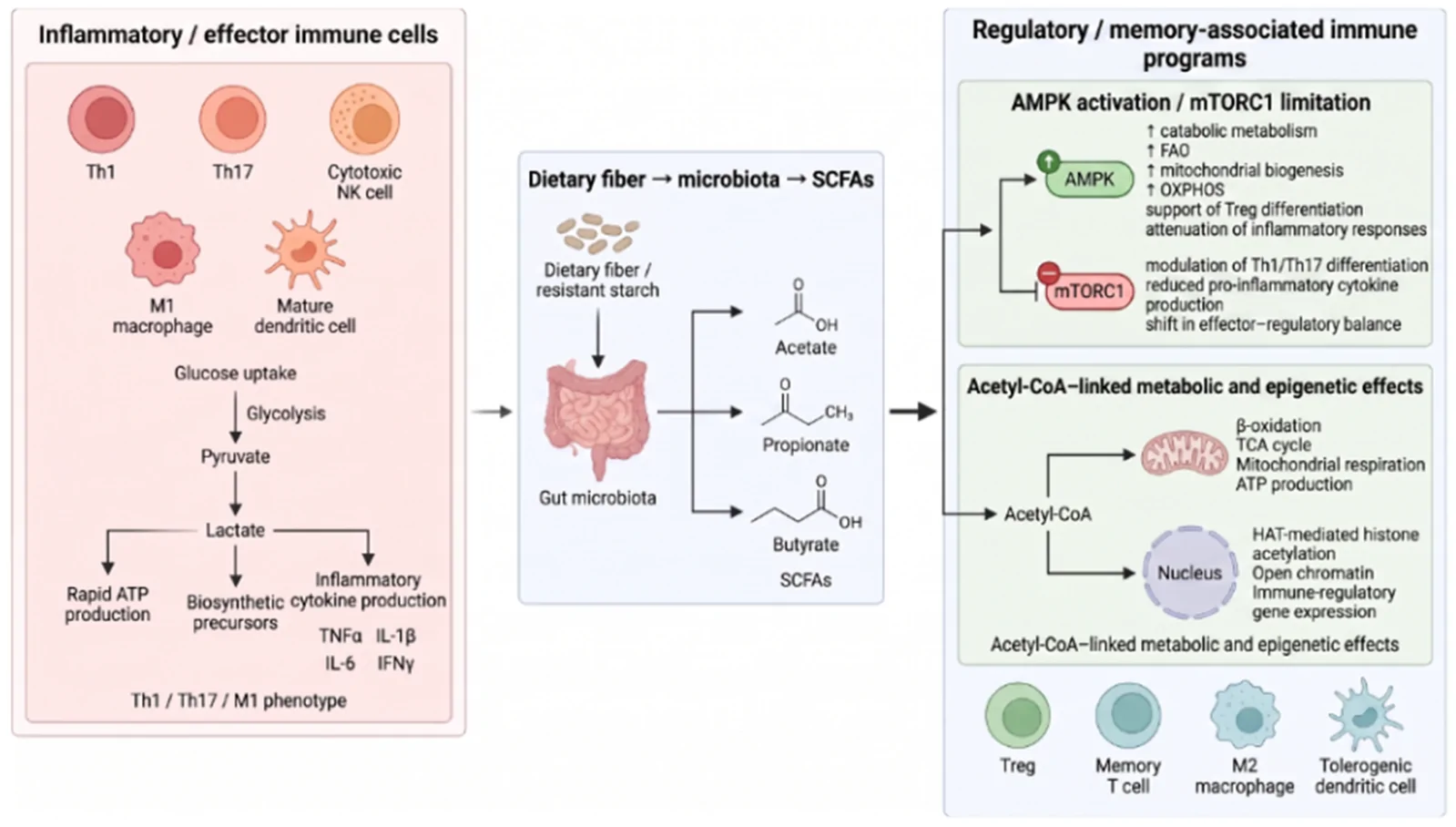
SCFA-associated modulation of immune-cell immunometabolism [70,71,72,73,74,75,76]. SCFAs influence metabolic and epigenetic pathways involving AMPK, mTORC1, and acetyl-CoA, thereby modulating inflammatory, regulatory, and memory-associated immune programs in a context-dependent manner. Black arrows indicate the direction of the proposed metabolic or signaling effects; ↑ indicates an increase or activation, whereas the red minus symbol indicates inhibition or limitation. Created in BioRender. Mertowski, S. (2026) https://BioRender.com/drcpafg (accessed on 10 July 2026).

SCFAs play a key role in regulating the metabolic balance described above [70]. In addition to their well-documented epigenetic effects, SCFAs also influence the activity of the cell’s main energy sensors, particularly AMP-activated protein kinase (AMPK) and the mTORC1 complex. AMPK functions as an intracellular energy sensor that, upon activation, orchestrates catabolic pathways to restore ATP homeostasis. Experimental studies indicate that butyrate and propionate can activate AMPK and promote metabolic changes associated with increased fatty acid oxidation and mitochondrial function [71]. Under these experimental conditions, such metabolic changes have been associated with the promotion of regulatory immune phenotypes and attenuation of inflammatory responses [72]. In contrast, the mTORC1 pathway integrates signals related to nutrient availability and stimulates anabolic processes. Excessive mTOR activation promotes the differentiation of effector lymphocytes, particularly the Th1 and Th17 populations, which are responsible for the development of many autoimmune diseases [73]. A growing body of evidence suggests that SCFAs can indirectly inhibit mTORC1 activity, thereby restoring the balance between effector and regulatory cells. Experimental evidence suggests that modulation of mTORC1 signaling by SCFAs may contribute to reduced pro-inflammatory cytokine production and attenuation of excessive immune-cell activation [70]. Another key aspect of SCFA function is their direct incorporation into the cell’s central metabolic pathways. After entering the cytoplasm, acetate and butyrate are converted to acetyl-CoA, which then feeds into the tricarboxylic acid (TCA) cycle, increasing the production of reduced cofactors used by the mitochondrial respiratory chain. In this way, SCFAs serve not only as signaling molecules but also as energy substrates that support immune cell function [74]. Crucially, acetyl-CoA serves as a critical metabolic bridge intersecting cellular bioenergetics with epigenetic regulation. A portion of the mitochondrial acetyl-CoA pool can be transported to the cell nucleus, where it serves as an acetyl group donor for histone acetyltransferases [75]. As a result, SCFA metabolites directly influence the degree of histone acetylation and the accessibility of chromatin to transcription factors [76]. This mechanism is one of the most compelling explanations for how bacterial metabolites can regulate the expression of genes responsible for maintaining immune tolerance and suppressing chronic inflammation Figure 4 [72].

### 4.3. The Effect of SCFAs on the Metabolic Profiles of Specific Leukocyte Populations

#### 4.3.1. Regulatory T Cells (Tregs)

**Figure 5:**
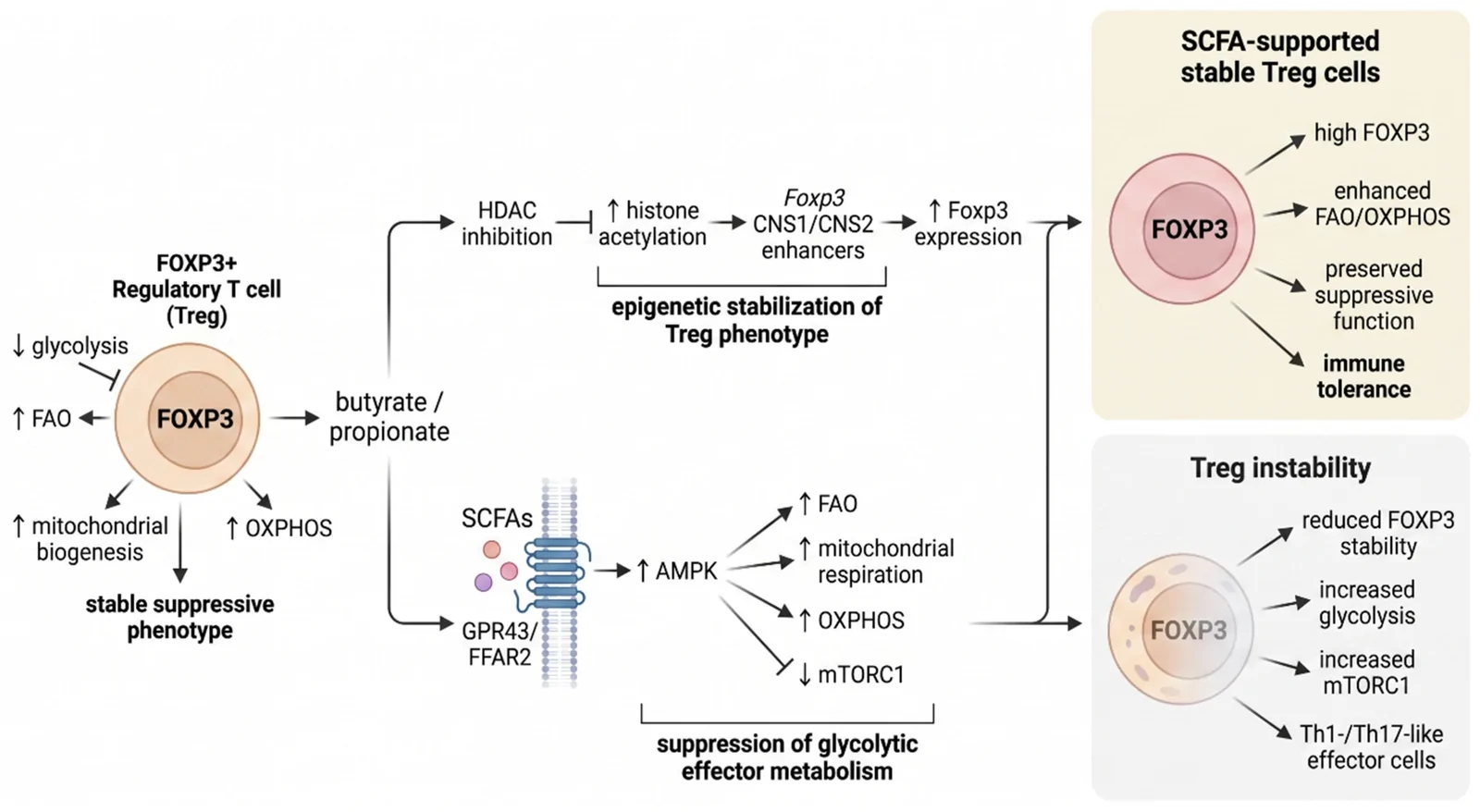
SCFA-mediated regulation of Treg metabolism and phenotype [37,68,69,70,71,72,73,74,75,76]. SCFAs, particularly butyrate and propionate, can support Treg-associated epigenetic and metabolic programs through HDAC inhibition and GPR43/FFAR2–AMPK signaling, thereby influencing FOXP3 expression, oxidative metabolism, and immune tolerance. ↑ indicates an increase; ↓ indicates a decrease. Created in BioRender. Mertowski, S. (2026) https://BioRender.com/drcpafg (accessed on 10 July 2026).

In Treg cells, the transcription factor FOXP3 is a key regulator of metabolism. Its expression leads to direct inhibition of glycolytic pathways while simultaneously enhancing processes related to FAO and mitochondrial biogenesis [77]. A disruption of this metabolic adaptation can lead to a loss of Treg phenotypic stability and the transition of these cells toward more pro-inflammatory effector populations [78]. Experimental studies suggest that butyrate can promote Foxp3 expression through increased histone acetylation at regulatory regions of the *Foxp3* locus [76]. SCFA-dependent receptor signaling has also been associated with AMPK activation, enhanced fatty acid oxidation, and modulation of mTORC1 activity, metabolic features that may support Treg stability under selected experimental conditions Figure 5 [37].

#### 4.3.2. Conventional CD4+ T Cells (Th1 and Th17)

**Figure 6:**
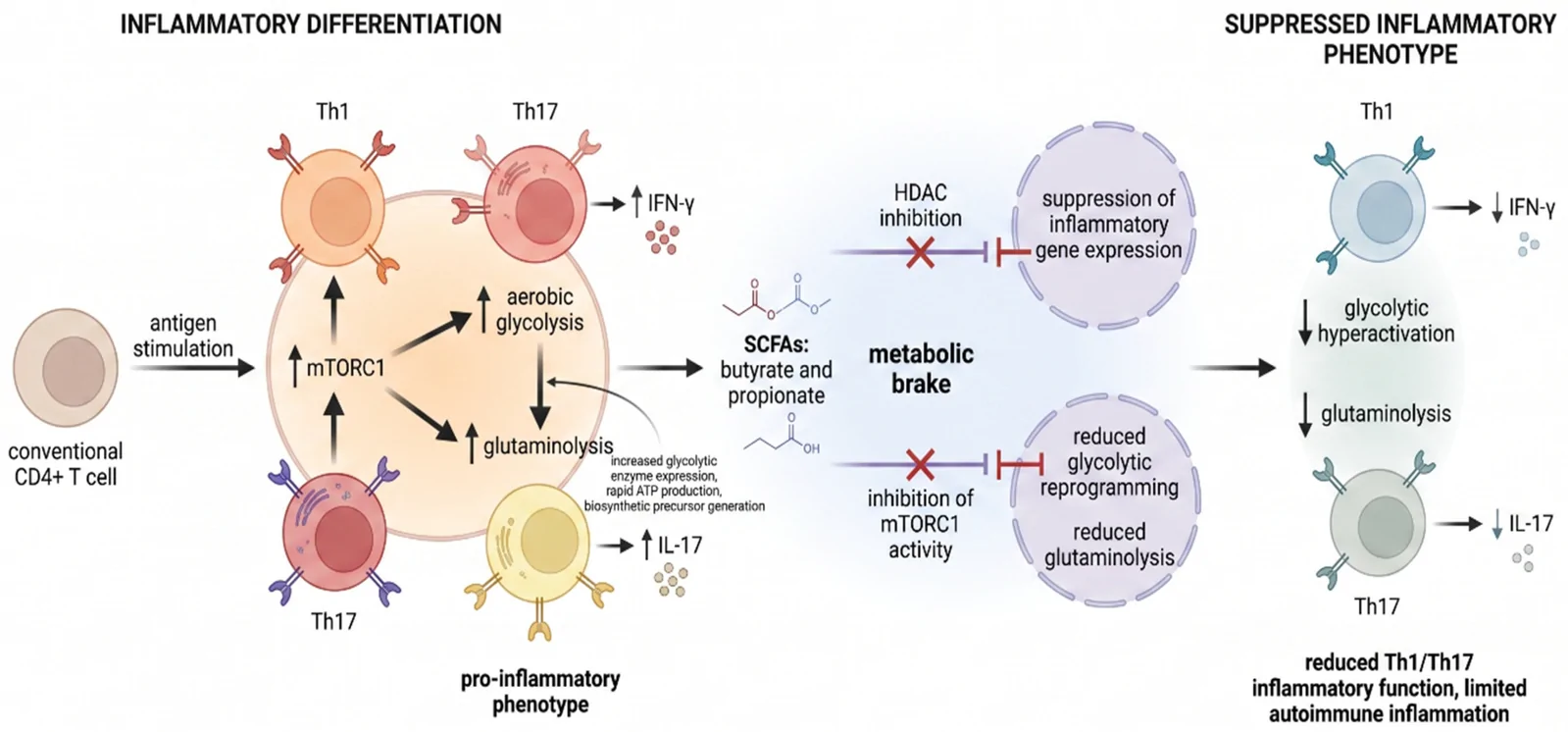
SCFA-mediated modulation of Th1 and Th17 immunometabolism [34,79,80,81,82,83,84,85]. SCFAs, particularly butyrate and propionate, can influence HDAC- and mTORC1-associated metabolic pathways, thereby modulating glycolysis, effector cytokine production, and Th1/Th17 inflammatory responses under selected conditions. ↑ indicates an increase; ↓ indicates a decrease. Created in BioRender. Mertowski, S. (2026) https://BioRender.com/drcpafg (accessed on 10 July 2026).

The metabolic profile of conventional helper T cells (Th1 and Th17) stands in stark contrast to that of regulatory T cells (Tregs) [79]. Following antigenic activation, these cells undergo rapid metabolic reprogramming, involving a shift in their bioenergetic phenotype toward intensive aerobic glycolysis and glutaminolysis [80]. The master regulator coordinating this process is mTORC1 kinase, which integrates microenvironmental signals and stimulates the transcription of glycolytic enzymes. The activation of glycolytic metabolism is an absolute prerequisite for these populations to perform their effector functions; as it has been demonstrated that blocking glycolysis directly impairs the ability of Th1 and Th17 lymphocytes to synthesize and secrete their signature pro-inflammatory cytokines—IFN-γ and IL-17, respectively [81,82]. Experimental evidence suggests that butyrate and propionate can modulate Th1- and Th17-associated metabolic programs through mechanisms involving HDAC activity and mTORC1 signaling [34,83]. Under these experimental conditions, SCFA exposure has been associated with reduced glycolytic activity and lower expression of selected effector cytokines. These findings suggest a potential mechanism through which SCFAs may attenuate Th1/Th17-driven inflammation, although the extent to which these effects translate to human autoimmune disease remains to be established Figure 6 [84,85].

#### 4.3.3. CD8+ T Lymphocytes (Effector and Memory)

**Figure 7:**
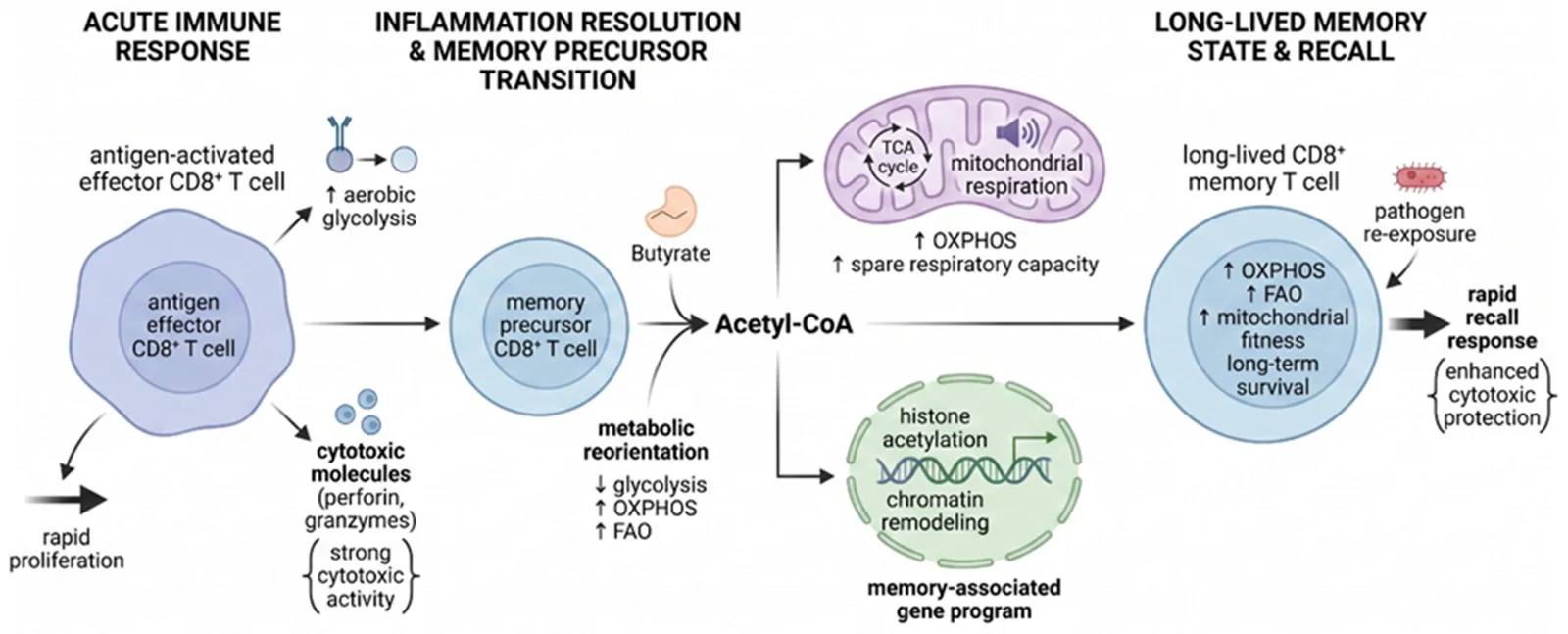
SCFA-associated metabolic and epigenetic programming of CD8+ memory T cells [37,56,86,87,88,89,90,91]. SCFAs, particularly butyrate, may support metabolic features associated with CD8+ memory T-cell differentiation through acetyl-CoA-dependent mitochondrial metabolism and epigenetic regulation. ↑ indicates an increase; ↓ indicates a decrease. Created in BioRender. Mertowski, S. (2026) https://BioRender.com/drcpafg (accessed on 10 July 2026).

In the population of CD8+, the relationship between functional status and bioenergetic profile is highly dynamic [86]. During the acute phase of the immune response, effector CD8+ T cells rely heavily on aerobic glycolysis to meet the intense biosynthetic and bioenergetic demands of clonal expansion and the secretion of lytic molecules, such as perforins and granzymes. However, as the inflammatory response subsides, the fraction of cells differentiating into long-lived memory T cells rapidly reorients its metabolism [87,88]. As with Treg cells, CD8+ memory cells abandon their glycolytic phenotype in favor of OXPHOS and FAO, a key requirement for long-term survival in peripheral tissues [89]. Experimental and mechanistic studies suggest that SCFAs, particularly butyrate, can contribute to the metabolic and epigenetic programming associated with CD8+ memory T-cell differentiation [90,91]. Once it enters the cell, exogenous butyrate is converted to acetyl-CoA and directly feeds into the mitochondrial tricarboxylic acid cycle [56]. At the same time, excess acetyl-CoA contributes to the remodeling of chromatin architecture in the cell nucleus, inducing specific epigenetic changes. Under experimental conditions, exposure to SCFAs has been associated with metabolic features compatible with memory T-cell formation, including enhanced mitochondrial fitness and respiratory capacity Figure 7 [37,91].

#### 4.3.4. B Lymphocytes and Plasma Cells

**Figure 8:**
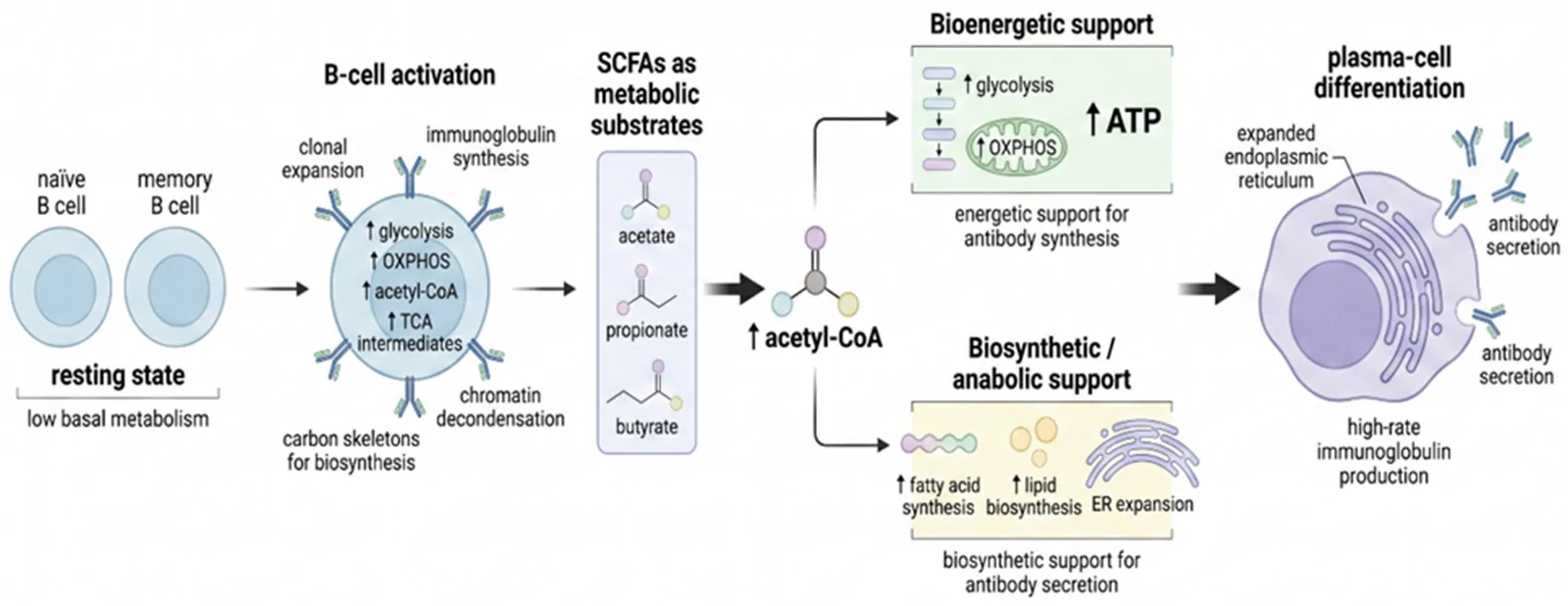
SCFA-associated metabolic regulation of B cells and plasma-cell antibody production [62,92,93,94]. SCFAs can influence B-cell bioenergetic and biosynthetic pathways by contributing to acetyl-CoA availability and modulating glycolysis, OXPHOS, and lipid metabolism, thereby supporting antibody production under selected experimental conditions. ↑ indicates an increase. Created in BioRender. Mertowski, S. (2026) https://BioRender.com/drcpafg (accessed on 10 July 2026).

A distinct metabolic dichotomy is observed within the B-cell lineage: quiescent populations, which include naive and memory B cells, are characterized by low, canonical bioenergetic activity, whereas their antigen-induced activation leads to a radical reprogramming of metabolic pathways [92]. Recognition of the antigen triggers a balanced, simultaneous increase in aerobic glycolysis and mitochondrial oxidative phosphorylation. Within the first 12 h after stimulation, this process leads to a significant increase in the intracellular acetyl-CoA pool. The intensification of metabolic processes and the accompanying influx of tricarboxylic acid cycle intermediates provide not only energy in the form of ATP, but also the carbon skeletons necessary for epigenetic chromatin decondensation, intense clonal proliferation, and massive immunoglobulin biosynthesis [93]. A key exogenous factor that modulates and supports this bioenergetic profile is SCFAs derived from the fermentation of dietary fiber by the gut microbiota. Experimental evidence suggests that SCFAs can contribute metabolic substrates to activated B cells and influence intracellular acetyl-CoA availability [62]. SCFA exposure has also been associated with changes in glycolytic and mitochondrial metabolism that may support the increased bioenergetic and biosynthetic demands accompanying B-cell activation and antibody production [62]. In activated B cells and plasma cells, increased lipid biosynthesis contributes to endoplasmic reticulum expansion required for high-rate immunoglobulin synthesis; however, the extent to which SCFAs directly control this process remains dependent on the experimental context Figure 8 [94].

#### 4.3.5. Dendritic Cells (DCs)

**Figure 9:**
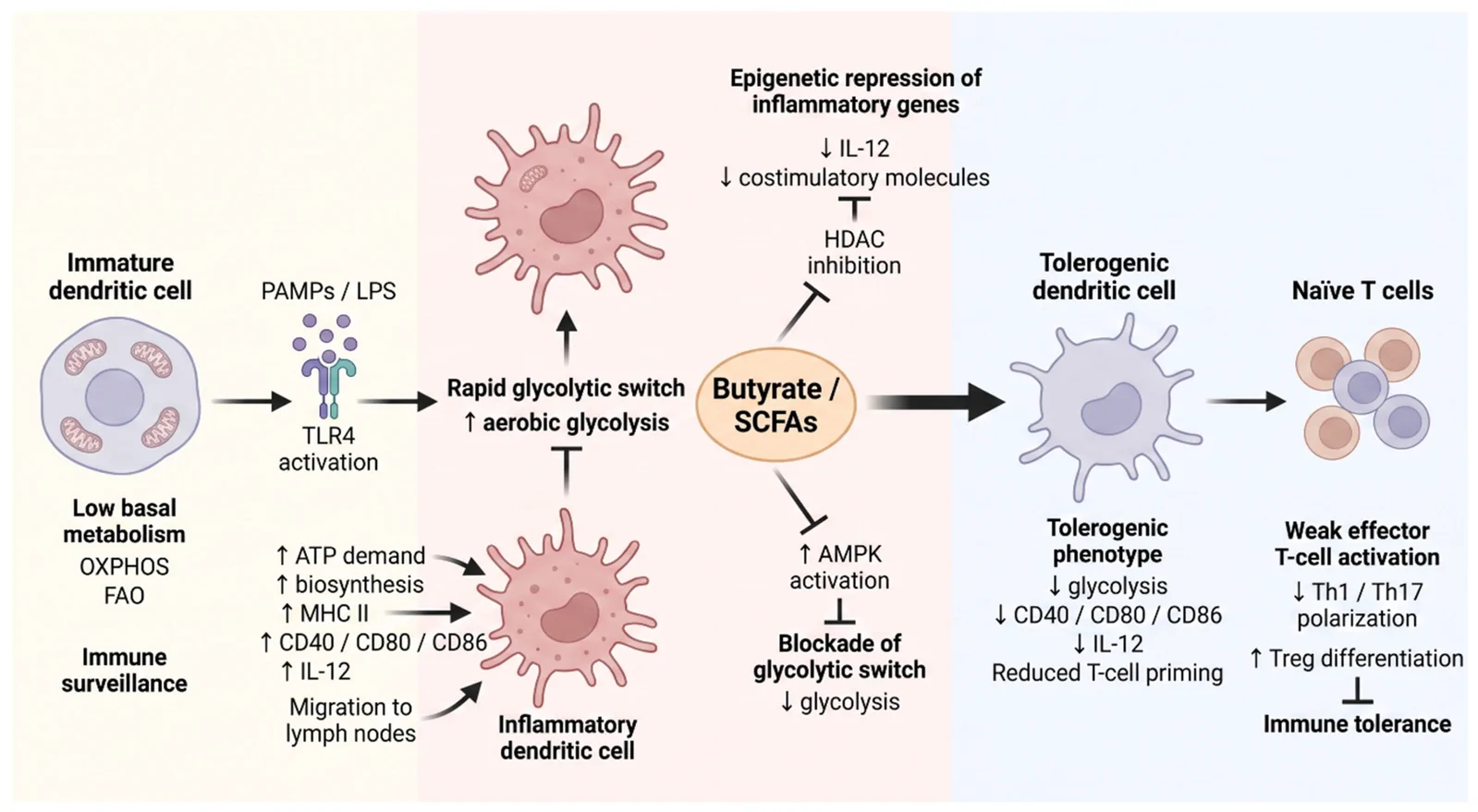
SCFA-associated modulation of dendritic-cell metabolic and functional programming [55,95,96,97,98,99,100,101]. SCFAs, particularly butyrate, can influence HDAC- and AMPK-associated pathways, thereby modulating dendritic-cell metabolism, costimulatory signaling, cytokine production, and T-cell priming toward a more tolerogenic phenotype under selected experimental conditions. ↑ indicates an increase; ↓ indicates a decrease. Created in BioRender. Mertowski, S. (2026) https://BioRender.com/drcpafg (accessed on 10 July 2026).

The influence of the bioenergetic profile on functional plasticity is also clearly evident in the case of DCs, which serve as a key bridge between nonspecific and specific immunity [95]. At rest, immature dendritic cells exhibit a relatively low metabolic rate based primarily on OXPHOS and FAO [96]. However, upon recognition of PAMPs—for example, through the activation of the TLR4 receptor—a rapid and radical shift in metabolism toward aerobic glycolysis occurs within just a few minutes. This immediate induction of the glycolytic pathway is absolutely essential for DCs to meet the enormous energy demands associated with their maturation [97]. It ensures the synthesis of structural proteins, the expression of major histocompatibility complex class II (MHC II) molecules and costimulatory receptors, and enables the dynamic migration of DCs to peripheral lymph nodes for effective antigen presentation [98]. In studies using human dendritic cells, butyrate has been shown to promote a more tolerogenic phenotype through mechanisms involving HDAC inhibition and GPR109A signaling. Experimental studies further suggest that SCFA-mediated modulation of HDAC and AMPK-related pathways can limit the glycolytic reprogramming associated with dendritic-cell activation [55]. They are characterized by a suppressed transcriptional and metabolic profile, reduced expression of costimulatory molecules, and inhibited secretion of pro-inflammatory cytokines (e.g., IL-12) [99,100]. Under these experimental conditions, SCFA-conditioned dendritic cells exhibit reduced pro-inflammatory cytokine and costimulatory signaling and an increased capacity to support regulatory rather than effector T-cell responses, Figure 9 [99,100,101].

#### 4.3.6. Natural Killer (NK) Cells

**Figure 10:**
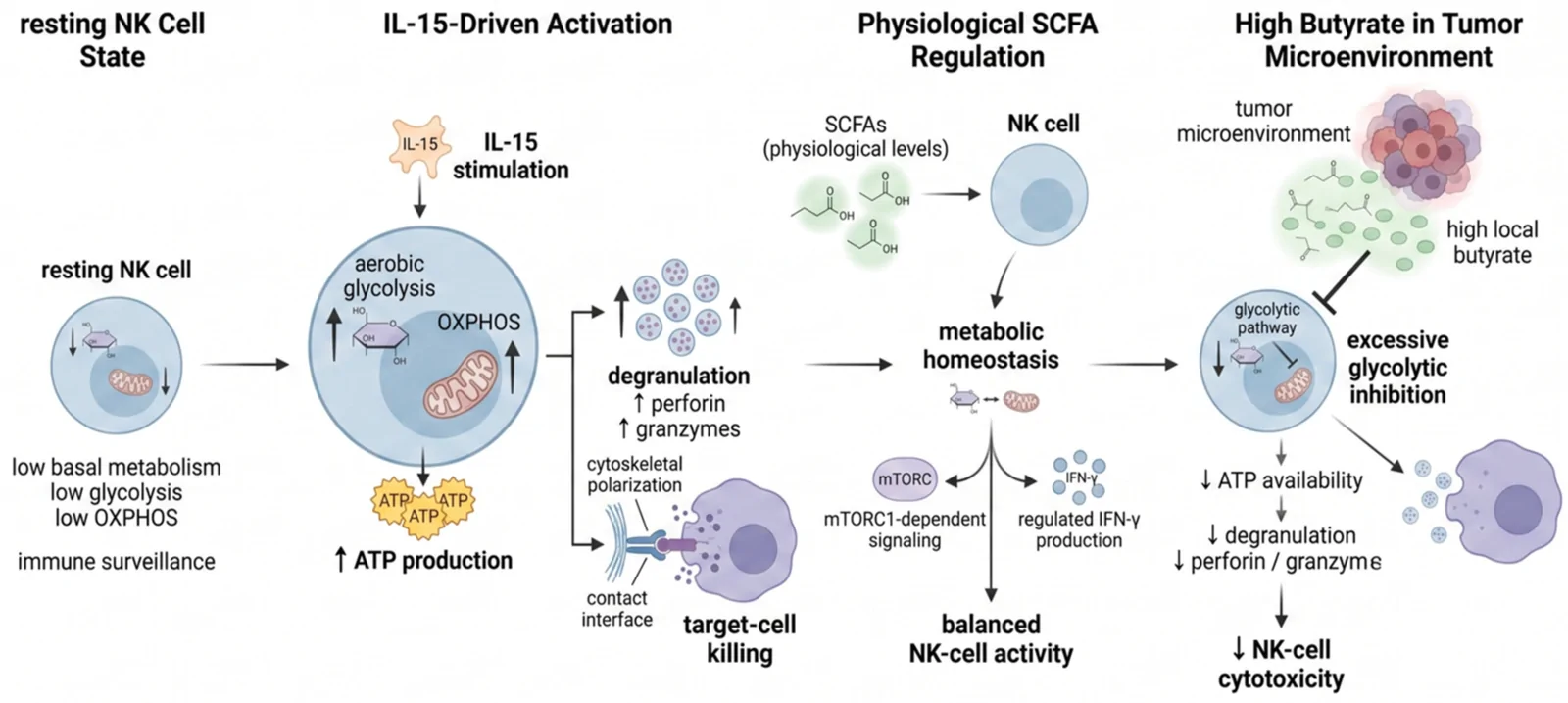
Context-dependent modulation of NK-cell immunometabolism and cytotoxicity by SCFAs [39,102,103,104,105,106,107,108]. SCFAs can influence NK-cell metabolic and effector programs, while high butyrate exposure may impair glycolysis, degranulation, and cytotoxicity under specific microenvironmental conditions. ↑ indicates an increase; ↓ indicates a decrease. Created in BioRender. Mertowski, S. (2026) https://BioRender.com/drcpafg (accessed on 10 July 2026).

The relationship between bioenergetic capacity and the ability to generate a rapid effector response is also clearly observed in NK cells, which are a key component of the nonspecific cellular immune response [102]. At rest, NK cells exhibit very low basal metabolic activity. However, in response to activating signals, such as cytokine stimulation with IL-15, there is a simultaneous and significant increase in both aerobic glycolysis and OXPHOS [103,104]. An exponential increase in ATP production is absolutely essential for NK cells to carry out the energy-intensive processes associated with their lytic function, including cytoskeletal polarization of surface receptors, degranulation, and the massive release of perforins and granzymes, which are necessary for the destruction of infected or cancerous cells [105,106]. The effect of short-chain fatty acids on NK cell biology exhibits unique plasticity and depends primarily on the microenvironmental context and the concentration of these metabolites. Studies of human NK cells indicate that butyrate can modify NK-cell metabolic and effector programs, including mTORC1-associated signaling and IFN-γ production [39,107]. In experimental studies using human NK cells, butyrate exposure was associated with reduced metabolic activity and impaired selected effector functions, indicating that its influence on NK-cell cytotoxicity may become suppressive under specific concentration and microenvironmental conditions [39,108]. These observations are also relevant to cancer biology, where microbiota-dependent modulation of cytotoxic T- and NK-cell function may contribute to variability in antitumor immune responses. More broadly, clinical evidence indicates that gut microbiome composition and function can influence the efficacy and toxicity of anticancer therapies, including chemotherapy, immunotherapy, and hematopoietic stem-cell transplantation, further emphasizing the clinical relevance of microbiome–immune interactions Figure 10 [109].

## 5. The Clinical Significance and Therapeutic Potential of SCFAs in Autoimmune and Autoinflammatory Diseases

The clinical discussion in this review focuses primarily on autoimmune and autoinflammatory diseases because these disorders provide a particularly relevant framework for examining the consequences of impaired microbiota–SCFA–immune interactions, including loss of immune tolerance, chronic inflammatory activation, altered T-cell and innate immune-cell function, and disruption of immunometabolic homeostasis. Although SCFA-dependent mechanisms such as HDAC inhibition, metabolic reprogramming, and modulation of NK- and T-cell activity are also relevant to cancer and other immune-mediated conditions, a comprehensive discussion of these fields would substantially broaden the scope of the present review. Cancer-related mechanisms are therefore addressed selectively where they illustrate the context-dependent effects of SCFAs, whereas the clinical section is intentionally focused on autoimmune and autoinflammatory disorders.

### 5.1. Etiopathogenic Differences Between Autoimmune and Autoinflammatory Processes

Although autoimmune and autoinflammatory diseases often share similar clinical features associated with chronic inflammation, their immunological mechanisms are different. n autoimmune diseases, dysregulation of adaptive immunity plays a central role and may lead to loss of tolerance to self-antigens. Consequently, this cascade drives the activation of autoreactive T and B lymphocytes, precipitates autoantibody production, and ultimately mediates host tissue destruction [2]. In contrast, autoinflammatory diseases result primarily from dysregulated activation of innate immune mechanisms [3]. Neutrophils, macrophages, and inflammasomes play a dominant role in these processes, as they are responsible for the excessive production of pro-inflammatory cytokines—primarily IL-1β and IL-18 [110]. Unlike autoimmune diseases, autoantibodies are not usually present in these conditions. Despite different mechanisms that trigger the disease process, a growing body of evidence suggests a possible common factor in both groups of conditions: disruption of the gut–immune axis [5]. Particular importance is attributed to gut dysbiosis and reduced production of SCFAs. In autoimmune diseases, SCFA deficiency primarily leads to impaired function of regulatory lymphocytes, whereas in autoinflammatory diseases, it mainly affects the activation of innate immune cells and the functioning of inflammasomes. In recent years, it has been increasingly emphasized that SCFAs serve as a bridge between the gut microbiota and the immune system. Their action involves regulating gene expression by inhibiting histone deacetylases and activating G protein-coupled receptors. As a result, these metabolites can modulate the function of many immune cell populations involved in both autoimmune and autoinflammatory processes [37].

### 5.2. The Effects of SCFAs on Selected Autoimmune Diseases

#### 5.2.1. Multiple Sclerosis

Multiple sclerosis (MS) is a chronic autoimmune disease of the central nervous system characterized by damage to the myelin sheaths of neurons [111]. The pathogenesis of this condition primarily involves Th1 and Th17 lymphocytes, as well as cytotoxic CD8+ T lymphocytes, which are capable of crossing the blood–brain barrier and initiating a local inflammatory process [112]. In recent years, there has been increasing evidence that patients with multiple sclerosis exhibit characteristic changes in the composition of their gut microbiota. These patients exhibit a reduction in the abundance of bacteria from the Lachnospiraceae and Firmicutes families, which are the primary producers of butyrate. As a result, there is a decrease in SCFA concentrations both in the intestinal lumen and in the peripheral circulation [113]. SCFA deficiency leads to a decrease in the number of Treg and Breg cells, which are physiologically responsible for maintaining immune tolerance by suppressing inflammatory processes [37]. Concurrently, aberrant Th17 hyperactivation and elevated pro-inflammatory cytokine secretion facilitate the trafficking of effector cells across the blood–brain barrier, culminating in central nervous system demyelination. Furthermore, activated T lymphocytes and antigen-presenting cells produce pro-inflammatory cytokines such as IFN-γ, TNF-α, IL-17, and GM-CSF, which can exacerbate the inflammatory process within the CNS [114]. In this disease, microglia and macrophages are activated, which can exacerbate damage to the myelin sheaths through the production of reactive oxygen species [115]. The most important risk factors for developing the disease are considered to be the presence of the HLA-DRB1*15:01 allele, as well as infection with the Epstein–Barr virus (EBV) and smoking [116].

#### 5.2.2. Type 1 Diabetes

Type 1 diabetes (T1D) is an autoimmune disease in which autoreactive T cells selectively destroy the β cells of the pancreatic islets. This chronic inflammatory process leads to progressive insulin deficiency and the development of hyperglycemia [117]. One of the mechanisms that may play a significant role in the pathogenesis of this disease is the gut–pancreas axis. In patients, as well as in individuals predisposed to developing the disease, there is a reduced number of bacteria responsible for the production of acetate and propionate [118]. A deficiency of these metabolites leads to impaired regulatory function and an exaggerated inflammatory response by the immune system [77]. CD8+ T cells play a major role in the autoimmune destruction of β cells; they recognize pancreatic autoantigens and induce apoptosis in insulin-producing cells. Furthermore, CD4+ T cells promote the development of an inflammatory response by secreting pro-inflammatory cytokines such as IFN-γ, TNF-α, and IL-1β [119]. The course of the disease is also characterized by the presence of autoantibodies directed against β-cell antigens, including glutamic acid decarboxylase (GAD65), insulin (IAA), tyrosine phosphatase IA-2, and the zinc transporter ZnT8 [120]. Although autoantibodies are not the direct cause of β-cell damage, they are an important marker of this disease. Genetic factors also play a significant role in the pathogenesis of T1D. The strongest association with the risk of developing the disease has been demonstrated for HLA class II genes, particularly HLA-DR3-DQ2 and HLA-DR4-DQ8, which influence the presentation of autoantigens to T cells. Environmental factors that may increase the risk of developing type 1 diabetes include Coxsackie B virus, vitamin D deficiency, and dietary habits during childhood [119]. The destruction of beta cells leads to insulin deficiency, which results in chronic hyperglycemia in the body. The consequences of this condition may include nephropathy, retinopathy, or the development of coronary artery disease [121].

#### 5.2.3. Rheumatoid Arthritis

Rheumatoid arthritis (RA) is a chronic autoimmune disease that leads to progressive joint damage. The pathogenesis primarily involves autoreactive T cells, as well as autoantibodies directed against the body’s own antigens. In patients with RA, a reduction in the number of butyrate-producing bacteria and an increase in the pro-inflammatory response are observed [122]. During the course of the disease, CD4+ T cells are activated, particularly the Th1 and Th17 subpopulations, which secrete numerous pro-inflammatory cytokines, such as TNF-α, IL-6, IL-17, and IFN-γ. These mediators stimulate the influx of inflammatory cells into the synovial membrane and sustain chronic inflammation [123]. B cells also contribute substantially by generating pathogenic autoantibodies, including rheumatoid factor (RF) and anti-citrullinated protein antibodies (ACPAs) [124]. Genetic factors, particularly HLA-DRB1 and HLA-DR4, also play a role in the pathogenesis of RA [125]. One of the most important environmental factors is smoking [118]. Reduced butyrate production leads to a compromise in the integrity of the intestinal barrier and impaired function of Tregs, which are responsible for maintaining immune tolerance. As a result, the autoimmune response is exacerbated and the production of pro-inflammatory cytokines is increased [126].

### 5.3. The Effects of SCFAs in Autoinflammatory Diseases

Unlike autoimmune diseases, in autoinflammatory diseases, SCFAs primarily affect the function of innate immune cells. This mainly involves the role of inflammasomes and the secretion of pro-inflammatory cytokines [36].

#### 5.3.1. Familial Mediterranean Fever

Familial Mediterranean fever (FMF) is a monogenic autoinflammatory disease associated with mutations in the MEFV gene, which encodes the pyrin protein. Pyrin plays a crucial role in regulating the innate immune response, as it is responsible for controlling inflammasome activation. Mutations in the MEFV gene abrogate this regulatory control, triggering the hypersecretion of key pro-inflammatory cytokines—primarily IL-1β and IL-18 [127]. Neutrophils play a key role in the pathogenesis of this condition. Their excessive activation triggers inflammation that affects the serous membranes, joints, and other organs [128]. Excessive activation of the inflammasome leads to increased caspase-1 activity, which is responsible for converting inactive cytokines into their biologically active forms. For this reason, a chronic inflammatory state persists in the patient’s body [129]. This condition in the body can lead to a serious complication known as AA amyloidosis, which develops as a result of prolonged elevated levels of amyloid A protein in the serum. This protein is a key acute-phase protein [130]. AA amyloidosis can lead to complications such as cardiomyopathy or hemorrhages, which may result in malabsorption or even death [131].

#### 5.3.2. Gout

Gout is an autoinflammatory disease caused by the deposition of sodium urate crystals in the joints [132]. These crystals are capable of activating the NLRP3 inflammasome. Activation of the NLRP3 inflammasome leads to the activation of caspase-1, which is responsible for converting inactive IL-1β and IL-18 precursors into their biologically active forms. These cytokines are essential for initiating and sustaining an acute inflammatory response in the body [133]. The recruitment of neutrophils to sites of monosodium urate (MSU) deposition drives the release of inflammatory mediators, reactive oxygen species, and lysosomal enzymes, the latter of which exacerbate local tissue destruction, pain, and edema [134]. One of the most significant factors in the development of the disease is hyperuricemia, a condition characterized by elevated levels of uric acid in the blood [133]. Other risk factors include, among others, a diet high in purines, obesity, and alcohol consumption. These factors cause the body to produce more uric acid or excrete less of it, which promotes the formation of sodium urate crystals [135]. Furthermore, these deposits do not accumulate only within the joints, but also in soft tissues and the kidneys. As a result, nephropathy may develop [136].

#### 5.3.3. Juvenile Idiopathic Arthritis

Juvenile idiopathic arthritis (JIA) is one of the most common chronic rheumatic diseases of childhood [137]. In many forms of the disease, there is an overactivation of the innate immune system, which involves the activation of macrophages and increased production of pro-inflammatory cytokines [138]. It is increasingly being noted that patients with this condition experience changes in their gut microbiota that lead to a decrease in SCFA production. A deficiency of these metabolites may contribute to the maintenance of a pro-inflammatory phenotype in effector cells and to chronic activation of the immune system. A key element of JIA pathogenesisis an imbalance between the pro-inflammatory and anti-inflammatory mechanisms of the immune system. During the course of the disease, increased activity of Th1 and Th17 lymphocytes and excessive production of pro-inflammatory cytokines, such as TNF-α, IL-1β, IL-6, and IL-17, are observed [139]. These mediators enhance the recruitment of inflammatory cells to the synovial membrane, leading to the persistence of chronic inflammation. At the same time, the function of Treg cells—which, under physiological conditions, are responsible for controlling the immune response and maintaining tolerance to self-tissues—is impaired. As a result, the inflammatory process becomes self-perpetuating, which promotes the development of joint damage. Prolonged activation of immune system cells leads to the proliferation of synovial cells and the formation of what is known as an inflammatory pannus [140]. This structure infiltrates the cartilage and subchondral bone, contributing to their gradual degradation. In addition, pro-inflammatory cytokines stimulate the activity of osteoclasts responsible for bone resorption, which can lead to bone erosions, joint deformities, and permanent impairment of joint function [141].

### 5.4. Inflammatory Bowel Diseases as a Model of Conditions with Mixed Etiopathogenesis

Inflammatory bowel diseases primarily include Crohn’s disease and ulcerative colitis. These conditions are a unique example of diseases because they involve mechanisms characteristic of both autoimmunity and autoinflammation. One of the most commonly reported abnormalities is a reduction in the abundance of butyrate-producing bacteria, particularly *Faecalibacterium prausnitzii* [142]. This leads to a decrease in local SCFA concentrations, impaired intestinal barrier function, and increased epithelial permeability [18]. SCFA deficiency results in both abnormal activation of macrophages and neutrophils and impaired Treg cell differentiation. Consequently, chronic inflammation persists, and progressive damage to the intestinal wall occurs. Additionally, an abnormal immune response directed against antigens present in the intestinal lumen plays a significant role in the pathogenesis of inflammatory bowel diseases [143]. In these patients, excessive activation of dendritic cells, macrophages, and effector T cells is observed, leading to increased production of pro-inflammatory cytokines such as TNF-α, IL-1β, IL-6, IL-12, IL-17, and IL-23 [144]. These cytokines sustain the inflammatory process, increase the influx of immune cells into the mucosa, and contribute to intestinal tissue damage. As the disease progresses, there is also an imbalance between effector lymphocytes and Tregs, which are responsible for maintaining immune tolerance. Impaired Treg function contributes to the persistence of inflammation and the uncontrolled activation of the immune response. The prolonged persistence of these abnormalities leads to chronic damage to the mucosa, the formation of ulcers, and impaired intestinal regeneration [145]. In Crohn’s disease, transmural inflammation spans the entire thickness of the gastrointestinal wall, frequently inducing fistulas and strictures [146]. Conversely, in ulcerative colitis, the inflammatory cascade remains restricted primarily to the colorectal mucosa and submucosa of the colon, leading to extensive damage to the epithelium and the formation of ulcers [147].

## 6. Therapeutic Strategies and Future Prospects

### 6.1. Rationale for Targeting the Microbiota–SCFA–Immune Axis

**Figure 11:**
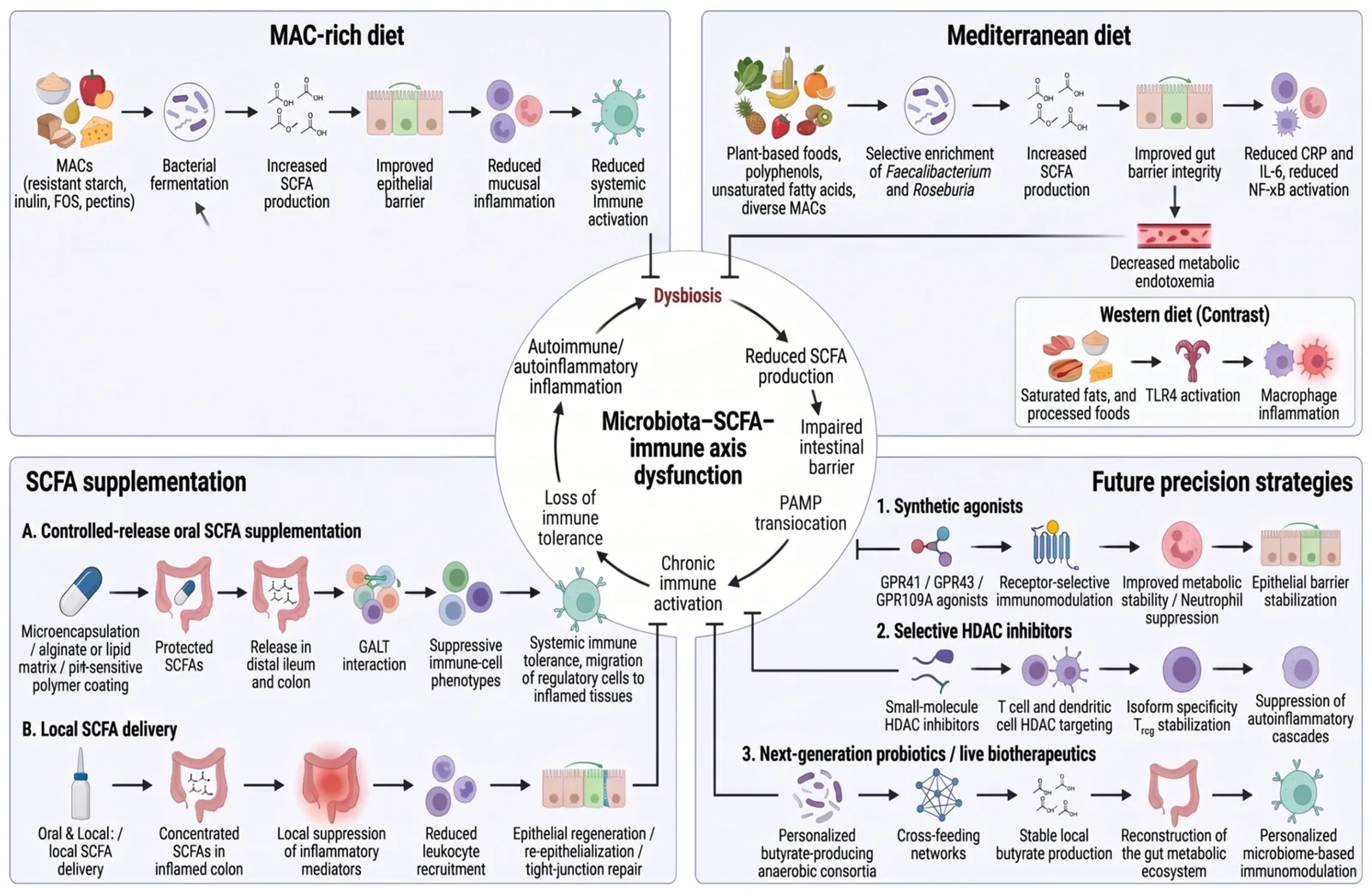
Therapeutic strategies targeting the microbiota–SCFA–immune axis [34,37,57,71,107,148,149,150,151,152,153,154,155,156,157,158,159,160,161,162,163,164,165,166,167,168,169,170,171,172,173,174,175,176,177,178,179,180,181,182,183,184,185,186,187,188,189]. Dietary approaches, SCFA supplementation, receptor- and HDAC-targeted strategies, and next-generation probiotics may modulate SCFA availability, epithelial barrier function, and immune responses. These approaches remain heterogeneous in their level of clinical validation and should be interpreted as complementary or emerging therapeutic strategies. Created in BioRender. Mertowski, S. (2026) https://BioRender.com/drcpafg (accessed on 10 July 2026).

In recent years, it has become increasingly clear that disruptions in the microbiota-SCFA-immune system axis are one of the key factors in the pathogenesis of autoimmune and autoinflammatory diseases [148]. Progressive dysbiosis, reduced SCFA production, and intestinal barrier dysfunction lead to a loss of immune tolerance and chronic activation of effector cells [149]. Understanding these relationships enables the development of therapeutic strategies that are not limited to conventional, non-specific immunosuppression—which carries a risk of systemic complications—but aim to restore the host’s immune homeostasis [150]. In this context, the gut–metabolic axis has transcended theoretical biology, emerging as a tangible cornerstone of translational medicine [151]. Current therapeutic paradigms focus on translating this potential into actionable clinical protocols by designing interventions that increase endogenous SCFA levels or precisely mimic their receptor-mediated and epigenetic effects, thereby enabling the safe resolution of the inflammatory response in both systemic and localized diseases Figure 11 [34,152].

### 6.2. Dietary Modulation as a Primary Therapeutic Strategy

#### 6.2.1. Carbohydrates Available to the Microbiota

Microbiota-accessible carbohydrates (MAC) serve as the primary substrate for bacterial fermentation, which leads to the production of SCFAs [177]. Unlike the traditional classification of dietary fiber into soluble and insoluble fractions, the MAC concept considers only those carbohydrates that are resistant to digestion by host enzymes but can be metabolized by specific bacterial taxa [178]. Studies have shown that a dietary deficiency of MAC leads to a decrease in the abundance of butyrate-producing bacteria, such as *Faecalibacterium prausnitzii*, *Roseburia* spp., and *Eubacterium rectale*, which correlates with increased inflammation and impaired immune tolerance [179]. Resistant starch is of particular importance among MACs; it includes several types that differ in their mechanism of resistance to digestion [180]. Resistant starch is categorized into four distinct types: RS1, a physically inaccessible starch trapped within intact whole grains; RS2, a naturally crystalline starch found in green bananas; RS3—retrograded starch, formed after cooking and cooling starchy products, which is one of the most readily fermentable sources of butyrate; and RS4—chemically modified starches, characterized by high resistance to digestive enzymes. Although all types function as MACs, RS2 and RS3 in particular strongly stimulate the growth of butyric acid-producing bacteria [181]. MAC also includes inulin and fructooligosaccharides (FOS), which have a strong bifidogenic effect, leading to the selective proliferation of bacteria of the genus Bifidobacterium. These bacteria produce lactate and acetate-key metabolites that are subsequently used by secondary fermentation bacteria to synthesize butyrate through cross-feeding [182,183]. From a translational perspective, increasing the dietary availability of specific MAC fractions may promote SCFA production and support epithelial barrier function; however, the magnitude of these effects varies according to baseline microbiota composition and the metabolic capacity of the host microbial community [57,184,185]. These observations provide a rationale for investigating MAC-based dietary interventions as complementary strategies for modulating inflammatory responses, although their clinical efficacy in autoimmune and autoinflammatory diseases remains insufficiently standardized [107,186,187].

#### 6.2.2. The Mediterranean Diet as a Dietary Model Aimed at Stimulating the SCFA-Immunity Axis

In studies examining the impact of dietary modifications on the course of immune-mediated diseases, particular attention is paid to the Mediterranean diet [188]. It is characterized by a high intake of unsaturated fatty acids, polyphenols, and various MAC fractions, while strictly limiting the consumption of red meat and highly processed foods [189]. The latest findings in the field of immunometabolism clearly indicate that high adherence to the Mediterranean diet correlates with increased intestinal production of SCFAs, which directly translates into a reduction in systemic inflammatory markers, such as C-reactive protein (CRP) and IL-6 [166]. The biochemical mechanism by which the Mediterranean diet stimulates SCFA synthesis is based on the synergistic interaction among its various components. Phytochemicals present in plant-based foods play a key role, particularly polyphenols (e.g., resveratrol and quercetin). These compounds are not fully absorbed in the small intestine and reach the colon, where they exert a prebiotic effect [167]. Studies based on 16S rRNA gene sequencing in patients following the MD model show selective proliferation of bacteria with high butyrate-producing potential, mainly from the genera *Faecalibacterium* and *Roseburia*, at the expense of bacteria with pro-inflammatory and pathogenic potential [168,169]. Furthermore, the high ratio of monounsaturated to saturated fatty acids in the Mediterranean diet is an important factor in modulating the intestinal microenvironment [167]. An excess of dietary saturated fatty acids (primarily palmitic acid)—a hallmark of the Western diet—acts as a direct ligand for TLR4, triggering an inflammatory cascade in macrophages and dendritic cells. Conversely, replacing these saturated lipids with monounsaturated oleic acid, concurrent with elevated acetate and propionate levels, suppresses the NF-κB signaling pathway [170,171]. From a clinical perspective, a dietary intervention based on the Mediterranean model helps restore the integrity of the intestinal barrier and reduce metabolic endotoxemia (the translocation of PAMPs into the portal circulation) [190]. This makes the Mediterranean diet a non-pharmacological, complementary component of therapy for patients suffering from chronic autoimmune and autoinflammatory diseases, capable of long-term reconfiguration of the host’s immunometabolic profile [172].

### 6.3. SCFA Supplementation: Characteristics of Administration Forms and Mechanisms of Immunomodulatory Action

Despite the high effectiveness of dietary interventions, in patients with established autoimmune and autoinflammatory diseases, modifying MAC intake alone often proves insufficient [191]. This is due to deep-seated, chronic dysbiosis, which leads to the loss of key bacterial taxa capable of carrying out efficient fermentation. In such cases, there is a clinical and immunological rationale for the direct administration of exogenous short-chain fatty acids as postbiotics [192]. However, the main barrier to conventional oral SCFA therapy is their pharmacokinetic properties. Free fatty acids, such as butyrate and propionate, when administered in their standard form, are absorbed almost immediately in the stomach and the initial segment of the small intestine [193]. For these metabolites to exert the desired effect on the immune system, it is necessary to use a variety of administration methods. The choice between controlled-release oral administration and topical application is not merely a matter of convenience, but a strategic decision that determines the spatial extent of the induced immune tolerance [173].

#### 6.3.1. Oral Controlled-Release Formulations

Modern pharmaceutical technology makes it possible to bypass the upper gastrointestinal tract barrier through advanced drug delivery systems, such as microencapsulation (in lipophilic or alginate matrices) and the use of pH-sensitive polymer coatings. These forms protect SCFA anions from premature absorption, ensuring their stable transit and release only in the distal ileum and colon, where they undergo enzymatic breakdown by the local microbiota [172,174,175,176]. From an immunological perspective, the goal of protected oral formulations is to produce a systemic effect [194]. Free metabolites released in the distal portions of the intestine interact directly with the cells of the GALT lymphoid tissue, which serves as the body’s primary center for programming immune tolerance [195]. The latest findings in the field of translational medicine show that such controlled, diffuse exposure reconfigures the host’s peripheral profile [196]. Upon stimulation within the SCFA-rich intestinal microenvironment, immune cells acquire a regulatory phenotype and egress from the mesenteric vasculature. Carried by the systemic circulation, they migrate to distant organs and tissues affected by the disease process [37,107]. It is precisely this mechanism of migration and systemic suppression of autoaggression that currently forms the basis of the latest research into the incorporation of propionate or butyrate supplementation into treatment protocols for systemic autoimmune diseases, such as MS [197]. Research on this form of administration confirms that oral administration of protected metabolites helps to suppress pathogenic inflammatory reactions in barrier structures and generate a distant neuroprotective effect in the central nervous system [198,199]. The protected oral formulation is therefore designed to restore global immunometabolic homeostasis over the long term and to inhibit autoimmune processes outside the gastrointestinal tract itself [107].

#### 6.3.2. Topical Forms of Administration

A completely different strategy is represented by topical administration, most commonly in the form of rectal suppositories. This form is administered to completely bypass the gastrointestinal tract and immediately deliver maximum, concentrated doses of SCFA solutions (or isolated butyrate) directly to the distal colon [200,201]. While the oral form aims to spread the tolerizing effect throughout the body, the goal of rectal infusions is to induce a strong, immediate local effect. This method of administration is justified in the acute phase of autoinflammatory diseases, in which there is a drastic disruption of the epithelial barrier and a violent inflammatory flare-up localized in the colon [202,203]. When administered topically, the free anion penetrates the lamina propria of the mucosa, directly suppressing the hyperactivity of the cells of the innate immune response located there. This blocks the synthesis of local inflammatory mediators, which rapidly reduces the influx of subsequent waves of effector leukocytes from the blood and halts the progressive destruction of intestinal wall tissues [156]. At the same time, the local presence of SCFAs acts as an immediate trophic factor that stimulates the regeneration and re-epithelialization of damaged epithelium. The rapid restoration of the intestine’s physical barrier function and the sealing of intercellular junctions prevent further penetration of toxins and antigens from the gastrointestinal lumen. This breaks the vicious cycle of inflammation, wherein epithelial hyperpermeability continuously drives local immune autoaggression [71,157]. Clinical studies have investigated topical SCFA administration, including butyrate-containing preparations, in patients with ulcerative colitis, with some studies reporting improvements in selected clinical or inflammatory outcomes [158,173]. However, the available evidence remains limited and heterogeneous, and these findings do not currently establish topical SCFA therapy as a standardized treatment for ulcerative colitis. Selected human studies evaluating SCFA-based interventions are summarized in Table 5, with particular emphasis on study population, SCFA formulation and route of administration, dose, treatment duration, clinical or immunological outcomes, reported adverse effects, and major methodological limitations.

### 6.4. Therapeutic Challenges and Future Prospects

Despite substantial experimental evidence supporting the immunometabolic effects of SCFAs, their translation into standardized clinical interventions remains challenging [170,188,189]. The therapeutic activity of SCFAs depends not only on the administered compound but also on its concentration, route of delivery, tissue exposure, host metabolic status, and baseline microbiota composition [13,170,188]. Moreover, much of the current mechanistic evidence derives from in vitro studies and animal models, whereas well-controlled human intervention studies remain comparatively limited and heterogeneous [170,189]. Consequently, the development of clinically applicable SCFA-based strategies requires careful consideration of pharmacokinetic limitations, interindividual variability, optimal dosing, delivery systems, and disease-specific efficacy [170,188,189].

#### 6.4.1. Key Challenges in SCFA-Based Therapies

One practical limitation of direct SCFA supplementation is the unfavorable organoleptic profile of some compounds, particularly butyrate, whose pungent odor and taste may reduce long-term treatment adherence [189]. More importantly, natural SCFAs have limited pharmacokinetic stability and undergo extensive intestinal and hepatic metabolism. Butyrate is extensively utilized by colonocytes, whereas propionate undergoes substantial hepatic metabolism, resulting in relatively low systemic exposure. Although acetate reaches the peripheral circulation more efficiently, achieving reproducible therapeutic concentrations of individual SCFAs in distant tissues remains challenging [13,170].

Clinical translation is further complicated by considerable interindividual variability in microbiota composition and function. Differences in the abundance of SCFA-producing bacteria, availability of microbiota-accessible carbohydrates, intestinal environmental conditions, dietary patterns, and host metabolic characteristics may influence both endogenous SCFA production and the biological response to dietary or microbiota-targeted interventions [190]. Human dietary intervention studies have demonstrated substantial interindividual differences in microbiome and immune responses, supporting the need to consider baseline microbiota characteristics when designing SCFA-oriented therapeutic strategies [190].

Additional uncertainties concern the optimal SCFA formulation, route of administration, dose, treatment duration, and target tissue exposure [170,189]. Local administration may be advantageous when the intended therapeutic target is the intestinal mucosa, whereas systemic immune-mediated diseases would require sufficient exposure beyond the gastrointestinal tract. However, concentrations that produce pronounced immunological effects in experimental systems may not necessarily correspond to physiologically achievable or clinically appropriate concentrations in humans [170]. This distinction is particularly important because SCFA effects are not uniformly anti-inflammatory but may vary according to metabolite identity, concentration, immune-cell type, activation status, and tissue microenvironment [34,39]. In human NK cells, for example, butyrate has been shown experimentally to impair selected metabolic and effector functions, illustrating that increased SCFA exposure cannot automatically be interpreted as therapeutically beneficial in every immunological context [39].

These limitations are compounded by the relatively restricted and heterogeneous clinical evidence base. Although human studies have evaluated dietary interventions, butyrate supplementation, and microbiota-directed strategies, considerable variation in patient populations, formulations, doses, treatment duration, and outcome measures limits direct comparison between studies and prevents the establishment of standardized therapeutic protocols [160,161,193]. Therefore, the clinical efficacy, long-term safety, optimal dosing strategies, and disease-specific indications for SCFA-based interventions require further validation in adequately powered and standardized clinical trials [160,193].

#### 6.4.2. Emerging Strategies: Receptor Targeting, Epigenetic Modulation, and Microbiota-Based Approaches

To overcome some of the pharmacokinetic limitations associated with natural SCFAs, several translational strategies are being investigated that aim to reproduce selected components of SCFA signaling without relying exclusively on direct metabolite supplementation [162,163,164]. One approach involves the development of synthetic small-molecule agonists targeting SCFA-responsive receptors, including FFAR2/GPR43, FFAR3/GPR41, and HCAR2/GPR109A [163,164]. Such compounds offer the theoretical advantage of greater receptor selectivity and more predictable pharmacological properties than endogenous SCFAs. Experimental evidence indicates that pharmacological modulation of these receptors may influence inflammatory signaling, metabolic regulation, and immune-cell activity [208,209]. However, receptor expression and functional responses vary among tissues and immune-cell populations, and the consequences of sustained receptor activation in humans remain incompletely characterized [164].

A second strategy involves targeting SCFA-sensitive epigenetic pathways. Butyrate and propionate can inhibit histone deacetylase activity and thereby modify chromatin accessibility and transcriptional programs involved in immune-cell differentiation and inflammatory regulation [34,153,165]. HDAC-targeted pharmacological approaches could theoretically reproduce selected transcriptional consequences of SCFA exposure with greater molecular selectivity. However, HDACs regulate numerous transcriptional programs in both immune and non-immune cells, and pharmacological inhibition may therefore produce broad biological effects [153,165]. The therapeutic relevance of this strategy will consequently depend on achieving sufficient isoform, cell-type, and tissue selectivity while limiting unintended effects.

Microbiota-based interventions represent another emerging therapeutic strategy. Next-generation probiotics (NGPs) and live biotherapeutic products are being developed using selected commensal microorganisms with defined metabolic properties, including taxa associated with butyrate production and other potentially beneficial metabolic functions [154,155,214,215]. Unlike conventional probiotic formulations based predominantly on broadly used *Lactobacillus* or *Bifidobacterium* strains, NGP approaches may include commensal species such as *Faecalibacterium prausnitzii* and other highly specialized intestinal microorganisms [154,155]. Advances in multi-omic profiling, microbial ecology, and synthetic biology may facilitate the selection or engineering of microbial strains and consortia with more predictable functional properties [154,214,216].

Nevertheless, their clinical translation remains challenging. Many candidate NGPs are strictly or extremely oxygen sensitive, complicating production, storage, formulation, and viable delivery to the colon [155]. In addition, therapeutic performance may depend on successful engraftment or functional persistence, ecological compatibility with the resident microbiota, availability of appropriate metabolic substrates, host diet, and individual colonization resistance [154,155,216]. Current reviews of programmable and next-generation probiotics therefore emphasize microbiome variability, manufacturing, delivery, safety, biocontainment, and regulatory complexity as important barriers to routine clinical implementation. Consequently, next-generation microbial consortia should currently be regarded as promising investigational tools rather than established substitutes for direct SCFA therapy [154,155,216]. Their future clinical implementation will require demonstration of reproducible functional activity, controlled metabolite production, long-term safety, and clinically meaningful efficacy in well-defined patient populations [155,214,216].

## 7. Conclusions

SCFAs represent key mediators of communication between the gut microbiota, cellular metabolism, and immune regulation. Their effects extend beyond the intestinal environment and involve receptor-dependent signaling, epigenetic regulation, and metabolic reprogramming of both innate and adaptive immune cells. Importantly, these effects are highly context-dependent and vary according to SCFA type, concentration, tissue microenvironment, immune-cell state, and disease setting. Although preclinical and emerging clinical evidence supports the therapeutic potential of targeting the microbiota–SCFA–immune axis, current human data remain heterogeneous and insufficient to establish standardized therapeutic approaches. Future studies should therefore focus on defining optimal formulations, doses, delivery strategies, and patient-specific factors that determine clinical responsiveness.

## Figures and Tables

**Table 1:** Comparative characteristics of the major SCFAs [16,33,34,35,36,37,38,39].

Feature	Acetate	Propionate	Butyrate
Carbon chain	C2	C3	C4
Relative abundance in the colon	Highest; approximately 60% of major SCFAs	Approximately 20%	Approximately 20%
Main metabolic fate	Enters peripheral circulation and contributes to systemic energy and lipid metabolism	Predominantly transported to the liver and used as a gluconeogenic substrate	Extensively oxidized by colonocytes as a major local energy source
Systemic availability	Highest among major SCFAs	Intermediate/low	Very low because of extensive colonic utilization and first-pass metabolism
Major receptor profile	Strong activity at FFAR2/GPR43; weaker at FFAR3/GPR41	Activates FFAR2/GPR43 and FFAR3/GPR41	Activates FFAR3/GPR41 and HCAR2/GPR109A; also interacts with FFAR2
HDAC-related activity	Relatively weak	Moderate	Strongest among the three major SCFAs
Predominant biological emphasis	Systemic metabolic signaling and immune modulation	Hepatic and immunometabolic regulation	Epithelial barrier support, local immunoregulation and epigenetic modulation
Context-dependent effects	Generally less potent as an HDAC modulator	May exert both metabolic and immunoregulatory effects depending on tissue and concentration	Strong local effects but may suppress selected effector functions, including NK-cell activity, at higher concentrations

**Table 2:** Selected receptors involved in short-chain fatty acid signaling and their immunological relevance [35,36,42,43,44].

Feature	FFAR2/GPR43	FFAR3/GPR41	HCAR2/GPR109A
Gene	*FFAR2*	*FFAR3*	*HCAR2*
Major SCFA ligands	Acetate and propionate; butyrate has lower activity	Propionate and butyrate; weaker response to acetate	Butyrate; also activated by niacin and D-β-hydroxybutyrate
Predominant expression relevant to immunity	Neutrophils, monocytes/macrophages, intestinal epithelial cells and other leukocyte populations	Intestinal, metabolic and neuroendocrine tissues; selected immune-cell populations	Intestinal epithelial cells, adipose tissue and selected innate immune cells, including neutrophils
Principal signaling	GPCR-mediated regulation of intracellular cAMP, Ca^2+^ and MAPK-related signaling	GPCR-mediated modulation of cAMP and metabolic/neuroendocrine signaling	Gi-coupled signaling with inhibition of cAMP and modulation of anti-inflammatory pathways
Major immunological relevance	Regulation of neutrophil recruitment, cytokine production, intestinal inflammation and resolution of inflammatory responses	Links microbial SCFA production with intestinal, metabolic and neuroimmune signaling	Supports epithelial barrier function and anti-inflammatory responses and contributes to regulation of neutrophil activity
Functional interpretation	Particularly relevant to acetate/propionate-dependent immune signaling	Particularly responsive to propionate and butyrate and involved in systemic metabolic signaling	Important mediator of selected immunoregulatory effects of butyrate

**Table 3:** Context-dependent determinants of the immunomodulatory effects of SCFAs.

Contextual Factor	Potential Effect on SCFA Activity	Representative Evidence	Reference
SCFA identity	Acetate, propionate, and butyrate differ in metabolism, systemic availability, receptor engagement, and biological potency	Acetate reaches the peripheral circulation more efficiently, whereas butyrate is preferentially utilized by colonocytes and exhibits stronger HDAC-inhibitory activity	[16,37]
Local concentration	Biological effects may vary according to local SCFA concentration; higher concentrations may exert stronger metabolic or epigenetic effects and may suppress selected effector functions	Butyrate can markedly suppress activated human NK-cell metabolism and effector function	[39]
Tissue/microenvironment	The direction of the immune response depends on the local inflammatory and metabolic environment	SCFAs may promote either effector or regulatory T-cell differentiation depending on the surrounding cytokine milieu	[45]
Immune-cell type	Different immune populations respond differently to individual SCFAs	Butyrate and propionate suppress DC-mediated antigen-specific CD8+ T-cell activation, whereas acetate does not show the same effect	[38]
Activation state	Activated immune cells have different metabolic requirements from resting cells, which modifies their response to SCFAs	Activated NK cells depend strongly on mTORC1-associated metabolic reprogramming, while butyrate can restrict this pathway and reduce effector activity	[39]
Receptor expression	Effects depend on the distribution and ligand specificity of FFAR2/GPR43, FFAR3/GPR41, and HCAR2/GPR109A	Different immune and epithelial populations express distinct SCFA receptors and therefore differ in responsiveness	[37]
Host metabolic status	SCFA effects interact with cellular pathways including mTOR, AMPK, acetyl-CoA metabolism, and HDAC activity	SCFAs can regulate T-cell differentiation through HDAC inhibition and the mTOR–S6K pathway	[45]
Disease/immunological context	The same SCFA-mediated pathway may favor immune tolerance in one setting while limiting desirable effector responses in another	Butyrate may support regulatory responses but can also suppress NK or CD8+ effector functions, with potential implications for antitumor immunity	[38,39]

**Table 4:** Selected histone deacetylases relevant to the epigenetic and immunomodulatory effects of short-chain fatty acids [46,47,48,49,50,51,52,53].

HDAC	Class	Predominant Localization/Regulatory Characteristics	Immunological Relevance	Relevance to SCFA-Mediated Effects
HDAC1	Class I	Predominantly nuclear; component of transcriptional corepressor complexes	Regulation of inflammatory gene expression, NF-κB signaling, immune-cell differentiation and tolerance	Butyrate and propionate-mediated HDAC inhibition may increase histone acetylation and modify transcription of inflammatory and regulatory genes
HDAC2	Class I	Predominantly nuclear; closely related functionally to HDAC1	Regulation of inflammatory transcription, cytokine responses and immune-cell activation	Represents an important target of SCFA-associated epigenetic modulation together with HDAC1
HDAC3	Class I	Nuclear and cytoplasmic; associated with transcriptional and metabolic regulation	Links inflammatory signaling with cellular metabolism and cytokine regulation	SCFA-mediated modulation of HDAC3 activity may contribute to interactions between metabolism, epigenetic regulation and inflammation
HDAC8	Class I	Nuclear and cytoplasmic	Regulation of transcription and selected inflammatory pathways	May contribute to SCFA-dependent chromatin remodeling, although its role is less extensively characterized than that of HDAC1–3
HDAC4	Class IIa	Shuttles between nucleus and cytoplasm; strongly regulated by phosphorylation	Involved in immune-cell differentiation and inflammatory signaling	Potential indirect contributor to SCFA-associated regulation of inflammatory transcription
HDAC5	Class IIa	Nuclear–cytoplasmic shuttling	Modulation of inflammatory and differentiation-associated transcriptional programs	May participate in SCFA-associated regulation of inflammatory and tolerogenic gene expression
HDAC7	Class IIa	Nuclear–cytoplasmic shuttling; prominent regulatory role in lymphocytes	Regulation of lymphocyte development, activation and inflammatory signaling	Potential mediator of SCFA-sensitive transcriptional programs in immune cells
HDAC9	Class IIa	Predominantly nuclear with multiple isoforms	Regulation of lymphocyte function, cytokine production and inflammatory responses	May contribute to SCFA-associated modulation of immune-cell transcription, although direct evidence is comparatively limited

**Table 5:** Selected human studies evaluating SCFA-based interventions [204,205,206,207,208,209,210,211,212,213].

Study	Population	Intervention/SCFA Formulation	Dose	Duration	Main Outcomes	Adverse Effects	Main Limitations
[204]	101 adults with clinically stable MS	Oral propionic acid as add-on therapy vs. placebo	500 mg twice daily	90 days	Significant reduction in serum neurofilament light chain; trend toward improvement in motor fatigue	AEs in 31% vs. 18% placebo; bloating significantly more frequent; no treatment-related serious AEs	Single-center phase 2b trial; short duration; biomarker primary endpoint rather than relapse/disability outcome
[205]	MS patients, including therapy-naïve and DMT-treated patients	Oral propionic acid supplementation	1 g/day	Short-term 14 days; longitudinal follow-up up to 3 years	↑ Tregs, ↓ Th1/Th17 after 2 weeks; post-hoc long-term analyses suggested reduced relapse rate, disability stabilization and reduced brain atrophy	No serious AEs reported; mild GI events reported in <5%	Proof-of-concept design; long-term clinical findings were post-hoc and not based on randomized placebo-controlled follow-up
[206]	98 adults with active mild-to-moderate UC	Microencapsulated sodium butyrate as add-on therapy vs. placebo	300 mg twice daily	8 weeks	Higher rates of clinical improvement, clinical remission and biochemical remission; endoscopic improvement reported; no clear advantage for complete endoscopic remission	AEs were prospectively monitored; no major safety concern reported in the publication	Add-on rather than monotherapy; short follow-up; modest sample size
[207,208]	36 adults with active UC	Oral sodium butyrate vs. rice-starch placebo	600 mg once daily	12 weeks	↓ fecal calprotectin, hs-CRP, ESR, NLR and partial Mayo score; improved sleep, QoL, anxiety/depression measures; altered circadian-clock gene expression	No significant adverse effects reported	Small single-center trial; multiple biochemical and patient-reported outcomes; same cohort reported in two publications
[209]	72 children/adolescents completing study; newly diagnosed UC or colonic CD	Oral sodium butyrate added to standard therapy vs. placebo	150 mg twice daily	12 weeks	No significant differences in remission rate, disease activity or fecal calprotectin	No adverse events reported	Pediatric heterogeneous IBD population; concomitant standard therapy; limited subgroup power
[210]	103 patients with distal/left-sided UC; 91 completed	Rectal mixed SCFA enema vs. placebo	100 mL twice daily: acetate 80 mM, propionate 30 mM, butyrate 40 mM	6 weeks	No significant overall benefit; response signal in patients with disease episode <6 months; adherence associated with response	Not clearly reported in abstract	Primary trial result negative; subgroup finding; adherence problems
[211]	47 patients with active distal UC	Mixed SCFA enema vs. butyrate alone vs. saline	60 mL twice daily: mixed SCFA 130 mM or butyrate 100 mM	8 weeks	DAI decreased in all groups without between-group difference; fewer affected segments after butyrate vs. placebo at week 8	Not clearly reported in abstract	Small sample; substantial placebo response; underpowered for primary endpoint
[212]	40 patients with mild-to-moderate distal UC	Rectal mixed SCFA enema vs. placebo	100 mL twice daily: acetate 80 mM, propionate 30 mM, butyrate 40 mM	6 weeks	More improvement in SCFA group; significant between-group differences mainly for bleeding, urgency and self-evaluation	Not clearly reported in abstract	Small sample; baseline imbalance in disease severity; only selected outcomes differed significantly
[213]	35 patients with UC in clinical remission	Rectal sodium butyrate vs. saline	60 mL of 100 mM sodium butyrate once daily	20 days	Only minor effects on inflammatory and oxidative-stress markers; ↑ IL-10/IL-12 ratio within butyrate group	Safety outcomes not sufficiently detailed in the abstract	Small sample; remission population; short intervention; biomarker-focused study

↑ indicates an increase; ↓ indicates a decrease.

## Data Availability

No new data were created or analyzed in this study. Data sharing is not applicable to this article.

## Abbreviations

The following abbreviations are used in this manuscript:

AA	Amyloid A/AA Amyloidosis
AMP	Adenosine Monophosphate
AMPK	AMP-activated Protein Kinase
APC	Antigen-Presenting Cell
ATP	Adenosine Triphosphate
BCFA	Branched-Chain Fatty Acid
cAMP	Cyclic Adenosine Monophosphate
CD4/CD8	Cluster of Differentiation 4/8
CD40/CD80/CD86	Cluster of Differentiation 40/80/86
CNS	Central Nervous System
CRP	C-reactive Protein
CSF	Colony-Stimulating Factor
CTBP1	C-Terminal Binding Protein 1
CXCR2	C-X-C Motif Chemokine Receptor 2
DNA	Deoxyribonucleic Acid
EAE	Experimental Autoimmune Encephalomyelitis
EBV	Epstein–Barr Virus
ERK	Extracellular Signal-Regulated Kinase
FAO	Fatty Acid Oxidation
FAS	Fatty Acid Synthase
FCN1	Ficolin-1
FFAR2/FFAR3	Free Fatty Acid Receptor 2/3
FMF	Familial Mediterranean Fever
FOS	Fructooligosaccharides
FOXP3	Forkhead Box P3
GAD65	Glutamic Acid Decarboxylase 65
GALT	Gut-Associated Lymphoid Tissue
GM-CSF	Granulocyte-Macrophage Colony-Stimulating Factor
GPCR	G-Protein Coupled Receptor
GPR41/GPR43/GPR109A	G-Protein Coupled Receptor 41/43/109A
HCAR2	Hydroxycarboxylic Acid Receptor 2
HDAC	Histone Deacetylase (HDAC1–9)
HIV	Human Immunodeficiency Virus
HLA	Human Leukocyte Antigen (HLA-DR3, HLA-DR4, HLA-DRB1)
IA-2	Islet Antigen-2
IAA	Insulin Autoantibodies
IBD	Inflammatory Bowel Disease
IFN	Interferon (IFN-γ)
IL	Interleukin (IL-1, IL-2, IL-6, IL-8, IL-10, IL-12, IL-15, IL-17, IL-18, IL-23)
JIA	Juvenile Idiopathic Arthritis
KAT5	Lysine Acetyltransferase 5
LPS	Lipopolysaccharide
MAC	Microbiota-Accessible Carbohydrates
MAC-rich	Microbiota-Accessible Carbohydrates-rich
MAPK	Mitogen-Activated Protein Kinase
MCT/MCT1	Monocarboxylate Transporter/1
MEF2C/MEF2D	Myocyte Enhancer Factor 2C/2D
MEFV	Mediterranean Fever Gene (Pyrin encoding gene)
MHC	Major Histocompatibility Complex
MS	Multiple Sclerosis
MSU	Monosodium Urate
mTORC1	Mechanistic Target of Rapamycin Complex 1
NCOR	Nuclear Receptor Co-repressor
NET	Neutrophil Extracellular Trap
NF-κB	Nuclear Factor kappa B
NK	Natural Killer (cells)
NLRP3	NOD-, LRR- and pyrin domain-containing protein 3
NLS	Nuclear Localization Signal
OXPHOS	Oxidative Phosphorylation
PAMP	Pathogen-Associated Molecular Pattern
PBMC	Peripheral Blood Mononuclear Cell
PRRs	Pattern-Recognition Receptors
RA	Rheumatoid Arthritis
RARA	Retinoic Acid Receptor Alpha
RCOR	REST Corepressor
RF	Rheumatoid Factor
ROR	Retinoic Acid Receptor-Related Orphan Receptor
S6K	Ribosomal Protein S6 Kinase
SCFA	Short-Chain Fatty Acid
SIN3	SIN3 Transcription Regulator
SLC16A1/SLC5A8/SLC5A12	Solute Carrier Family 16 Member 1/5 Member 8/5 Member 12
SMCT1/SMCT2	Sodium-Coupled Monocarboxylate Transporter 1/2
SMRT	Silencing Mediator for Retinoid and Thyroid Hormone Receptors
STAT3	Signal Transducer and Activator of Transcription 3
T1D/T1DM	Type 1 Diabetes Mellitus
TCA	Tricarboxylic Acid (Cycle)
Th1/Th17	T Helper 1/17 (cells)
TLR4	Toll-Like Receptor 4
TM	Transmembrane Domain (TM1–TM7)
TNF	Tumor Necrosis Factor
Treg	Regulatory T (cells)
